# Fast and Rigorous Computation of Gene and Pathway Scores from SNP-Based Summary Statistics

**DOI:** 10.1371/journal.pcbi.1004714

**Published:** 2016-01-25

**Authors:** David Lamparter, Daniel Marbach, Rico Rueedi, Zoltán Kutalik, Sven Bergmann

**Affiliations:** 1 Department of Medical Genetics, University of Lausanne, Lausanne, Switzerland; 2 Swiss Institute of Bioinformatics, Lausanne, Switzerland; 3 Institute of Social and Preventive Medicine (IUMSP), Centre Hospitalier Universitaire Vaudois (CHUV), Lausanne, Switzerland; Microsoft Research, UNITED STATES

## Abstract

Integrating single nucleotide polymorphism (SNP) p-values from genome-wide association studies (GWAS) across genes and pathways is a strategy to improve statistical power and gain biological insight. Here, we present *Pascal* (Pathway scoring algorithm), a powerful tool for computing gene and pathway scores from SNP-phenotype association summary statistics. For gene score computation, we implemented analytic and efficient numerical solutions to calculate test statistics. We examined in particular the sum and the maximum of chi-squared statistics, which measure the strongest and the average association signals per gene, respectively. For pathway scoring, we use a modified Fisher method, which offers not only significant power improvement over more traditional enrichment strategies, but also eliminates the problem of arbitrary threshold selection inherent in any binary membership based pathway enrichment approach. We demonstrate the marked increase in power by analyzing summary statistics from dozens of large meta-studies for various traits. Our extensive testing indicates that our method not only excels in rigorous type I error control, but also results in more biologically meaningful discoveries.

## Author Summary

Genome-wide association studies (GWAS) typically generate lists of trait- or disease-associated SNPs. Yet, such output sheds little light on the underlying molecular mechanisms and tools are needed to extract biological insight from the results at the SNP level. Pathway analysis tools integrate signals from multiple SNPs at various positions in the genome in order to map associated genomic regions to well-established pathways, i.e., sets of genes known to act in concert. The nature of GWAS association results requires specifically tailored methods for this task. Here, we present *Pascal* (Pathway scoring algorithm), a tool that allows gene and pathway-level analysis of GWAS association results without the need to access the original genotypic data. *Pascal* was designed to be fast, accurate and to have high power to detect relevant pathways. We extensively tested our approach on a large collection of real GWAS association results and saw better discovery of confirmed pathways than with other popular methods. We believe that these results together with the ease-of-use of our publicly available software will allow *Pascal* to become a useful addition to the toolbox of the GWAS community.

## Introduction

Genome-wide association studies (GWAS) have linked a large number of common genetic variants to various phenotypes. For most common phenotypes, high-powered meta-analyses have revealed tens to hundreds of single nucleotide polymorphisms (SNPs) with robust associations. However, deriving biological knowledge from these associations is often challenging[1,2]. Many genes function in multiple biological processes and it is typically not clear which of these processes is related to the phenotype in question.

Pathway analysis aims to provide insight into the biological processes involved by aggregating the association signal observed for a collection of SNPs into a pathway level signal. This is generally carried out in two steps: first, individual SNPs are mapped to genes and their association p-values are combined into gene scores; second, genes are grouped into pathways and their gene scores are combined into pathway scores. Existing tools vary in the methods used for each step and the strategies employed to correct for correlation due to linkage disequilibrium.

SNPs are usually mapped to genes based on physical distance[3], linkage disequilibrium (LD)[4], or a combination of both[5]. Genes are commonly assigned to pathways using well-established databases (such as Gene Ontology[6], KEGG[7], PANTHER[8], REACTOME[9], BIOCARTA[10]) or in-house annotation (based on co-expression[4], for example).

Various methods have been developed to aggregate SNP summary statistics into gene scores[3,11,12]. A common aggregation method is to use only the most significant SNP within a window encompassing the gene of interest, for example by assigning the maximum-of-chi-squares (MOCS) as the gene score statistic[3,13] (the contributing chi-squared values can be obtained from SNP p-values by using the inverse chi-squared quantile transformation). Another method is to combine results for all SNPs in the gene region, for example by using the sum-of-chi-squares (SOCS) statistic[14]. Both the MOCS and SOCS statistics are confounded by several properties of the gene. Specifically, in both cases it is important to correct for gene size and LD structure to obtain a well-calibrated p-value for the statistic. In the remainder of this paper, we also refer to the p-values of the MOCS and the SOCS statistics as *max* and *sum gene scores*, respectively. P-values can be estimated by phenotype label permutation, but this method is both computationally intensive and requires access to genotype data of the actual study, which are rarely shared[15]. Thus, one often has access only to association summary statistics and not the individual genotypes. In this case, one method is to regress out confounding factors[3]. This approach is employed in the popular MAGENTA tool, but provides only a partial solution as substantial residual confounding still remains[3].

An alternative approach, which we take here, is to exploit the fact that the null distributions of the MOCS and SOCS statistics depend solely on the pairwise correlation matrix of the contributing genotypes. In the absence of the original genotypes, this correlation matrix can still be estimated from ethnicity-matched, publicly available genotypic data, as has been proposed by us and others for conditional multi-SNP analysis of GWAS results[16,17]. This approach has been implemented in the *Versatile Gene-based Association Study* (VEGAS) software and yields results close to those from phenotype label permutation[11]. However, while VEGAS is faster than estimation via phenotype label permutation, it still relies on a Monte Carlo method for estimating the p-values. This limits its efficiency for highly significant gene scores.

Once gene scores have been computed, pathway analysis tools use various strategies to aggregate them across sets of related genes. The most common approach used for analysing GWAS meta-analysis results, as exemplified by the popular GWAS pathway analysis tool MAGENTA, is based on binary enrichment tests, which rely on a threshold parameter to define which genes are significantly associated with the trait[3,18]. However, with this strategy potential contributions of weakly associated genes that just missed the threshold are lost and there is no clear guidance on how the threshold parameter should be set. Indeed, it seems common practice to keep the default parameter without knowing whether other choices would produce better results[5].

**Fig 1:**
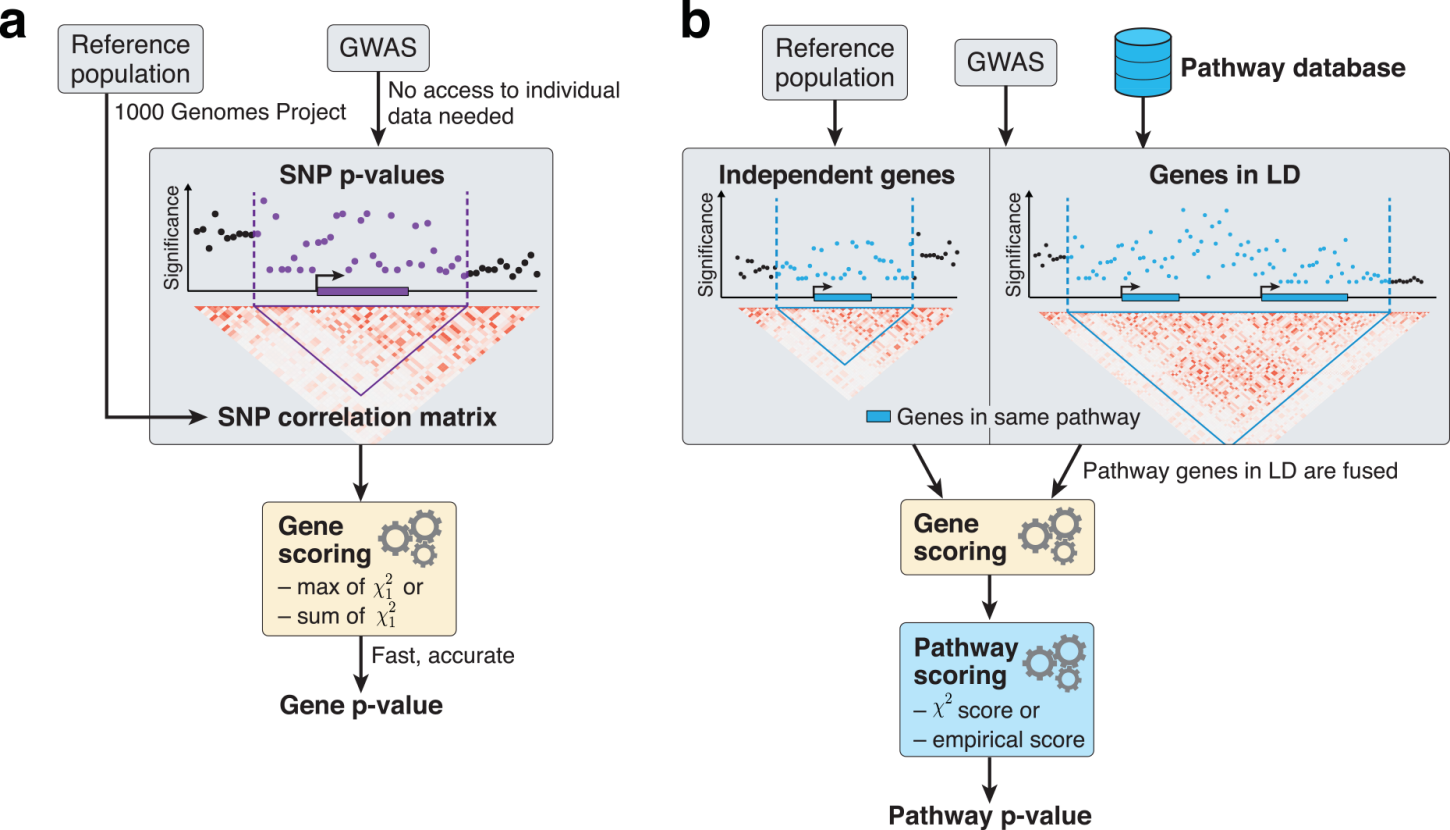
Overview of the methodology to compute gene and pathway scores. a) We compute gene scores by aggregating SNP p-values from a GWAS meta-analysis (without the need for individual genotypes), while correcting for linkage disequilibrium (LD) structure. To this end, we use numerical and analytic solutions to compute gene p-values efficiently and accurately given LD information from a reference population (e.g. one provided by the 1000 Genomes Project[22]). Two options are available: the max and sum of chi-squared statistics, which are based on the most significant SNP and the average association signal across the region, respectively. b) We use external databases to define gene sets for each reported pathway. We then compute pathway scores by combining the scores of genes that belong to the same pathways, i.e. gene sets. The fast gene scoring method allows us to dynamically recalculate gene scores by aggregating SNP p-values across pathway genes that are in LD and thus cannot be treated independently. This amounts to fusing the genes and computing a new score that takes the full LD structure of the corresponding locus into account. We evaluate pathway enrichment of high-scoring (possibly fused) genes using one of two parameter-free procedures (chi-squared or empirical score), avoiding any p-value thresholds inherent to standard binary enrichment tests.

In this work, we focus on improving two major aspects of pathway enrichment analysis (Fig 1). First, we incorporate numerical and analytic solutions for the p-value estimation of the MOCS and SOCS statistics. This removes the need for phenotype permutations or Monte Carlo simulations, thereby making the score computation faster. Second, we developed a rigorous type I error control strategy and implemented a modified Fisher method to compute parameter-free pathway scores[19]. While some elements of our algorithm have been proposed in other fields of statistical genetics[20,21], the novelty of our method lies in the unique combination of sophisticated analytical methods employed for pathway analysis, which results in improved computational speed, precision, type I error control and power.

In the following, we first evaluate the performance of our tool, demonstrating its speed gains and robust control of type I error. Then, using precision-recall analyses, comparing small to large GWAS results for lipid traits and Crohn’s disease, we demonstrate that our pathway scoring approach exhibits a gain in power compared to binary enrichment. Finally, we apply our method to dozens of large meta-analysis studies and evaluate power by counting the number of pathways passing the Bonferroni-corrected p-value threshold.

We provide this tool for gene and pathway scoring as a standalone, open-source software package called *Pascal*.

## Results

### *Pascal* computes genes scores rapidly and to very high precision

**Fig 2:**
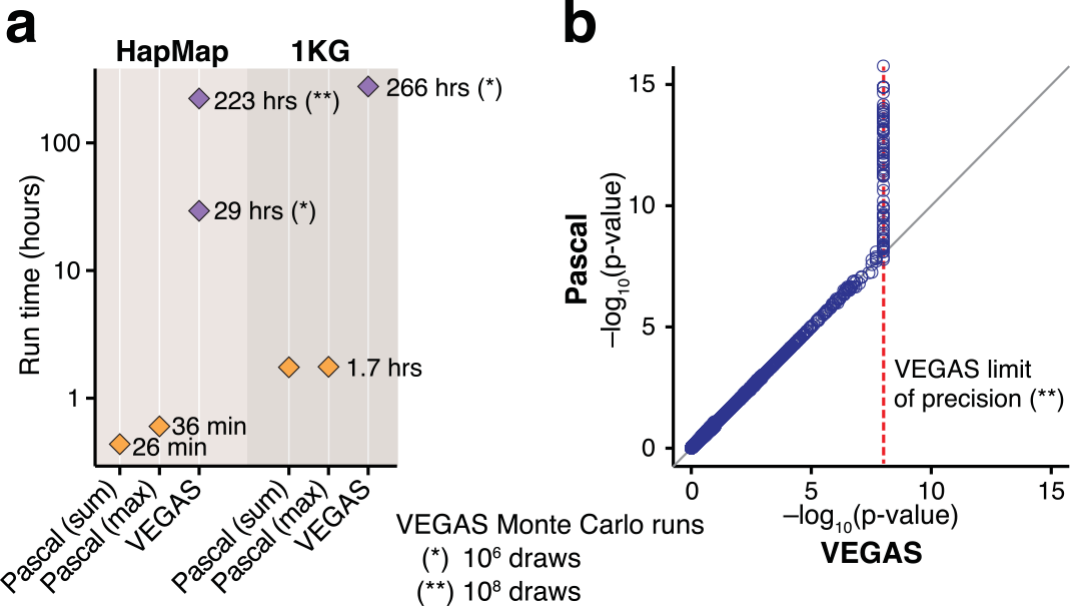
Comparing efficiency between VEGAS and *Pascal*. a) Run times of VEGAS and *Pascal* (both options). Gene scores were computed on two GWAS (one HapMap imputed[23], one 1KG imputed[22,25]) for 18,132 genes on a single core. *Pascal* was compared to VEGAS for the HapMap imputed study and VEGAS2 for the 1KG-imputed study. For this plot, VEGAS and VEGAS2 were used with the default maximum number of Monte Carlo samples of 10^6^ for both studies and additionally with 10^8^ Monte Carlo samples for the HapMap imputed study. b) Scatter plot of -log_10_-transformed gene p-values for the sum gene scores obtained by VEGAS and *Pascal*, respectively. P-values above 10^−6^ are in excellent concordance. Below this value VEGAS could not give precise estimates, since it was run with the maximal number of Monte Carlo samples set to 10^8^.

First, we compared the run time and precision of *Pascal* to those of VEGAS[11], one of the current state-of-the-art gene scoring tools. To this end, we applied both procedures to genome-wide p-values obtained from two large-scale GWAS meta-analyses: The first used about 2.5 million HapMap imputed SNPs[23,24] and the second was based on about 6.4 million SNPs imputed based on a common subset of 1000 Genomes Project (1KG) panel[22,25]. As benchmark we used the results from VEGAS for the former and VEGAS2 (a recent implementation of VEGAS that uses pre-computed LD matrices from 1KG[26]) for the latter. We observed a substantially smaller run time for our method in both cases (Fig 2A): for the HapMap imputed data, VEGAS took 29 hours to compute 18,132 gene scores, while *Pascal* was considerably faster, needing only about 30 minutes for either statistical test (sum or max) on a single core (Intel Xeon CPU, 2.8GHz). For the 1KG imputed data, *Pascal* finished the computation in under two hours for either statistic, whereas VEGAS2 took over ten days.

To compare the gene scores computed by the two methods, we increased the maximum number of Monte Carlo runs for VEGAS to 10^8^, at a high computational cost (about 9 days of runtime). We observed excellent concordance between the gene scores of *Pascal* and VEGAS, except for scores below 10^−6^: since we restricted VEGAS to 10^8^ Monte Carlo runs, it could not estimate p-values smaller than 10^−6^ with good precision (Fig 2B). In contrast, *Pascal* can compute gene scores with high precision for p-values down to 10^−15^. In summary, the analytic solutions incorporated in the *Pascal* algorithm offer a dramatic increase in efficiency and precision. Direct comparison of the sum and max gene scores of *Pascal* revealed good concordance between the two scoring methods. In cases where the results of two methods disagree, max scores tend to be more significant (S3 Fig).

The results reported here are all based on GWAS of European cohorts, thus we used the European panel from 1KG as reference panel. To evaluate whether this panel approximates LD matrices derived from other European cohorts sufficiently well, we compared results when using genotypes taken from the *CoLaus* cohort as reference panel[27]. We saw good concordance between the different reference panels for both the sum and the max gene scores for the largest HDL blood lipid GWAS to-date[23] (S2 Fig).

### *Pascal* controls for inflation due to neighbouring genes

In general, methods that compute pathway scores from gene scores assume independence of these scores under the null hypothesis. However, neighbouring genes often have correlated scores due to LD, and are sometimes part of the same pathway. This results in a non-uniform pathway score p-value distribution under the null hypothesis. MAGENTA deals with this problem by pruning gene scores based on LD and using only the highest gene score in the region. However, this introduces a bias toward high gene scores into the calculation of pathway scores[3].

Our fast gene score calculation allows us to address this issue with a gene-fusion strategy. In brief, for each pathway harbouring correlated genes, gene scores are recomputed jointly for each correlated gene set (i.e. fusion-gene) using the same method as for individual genes (Fig 1B, Methods), thus taking the full LD structure of the corresponding region into account.

**Fig 3:**
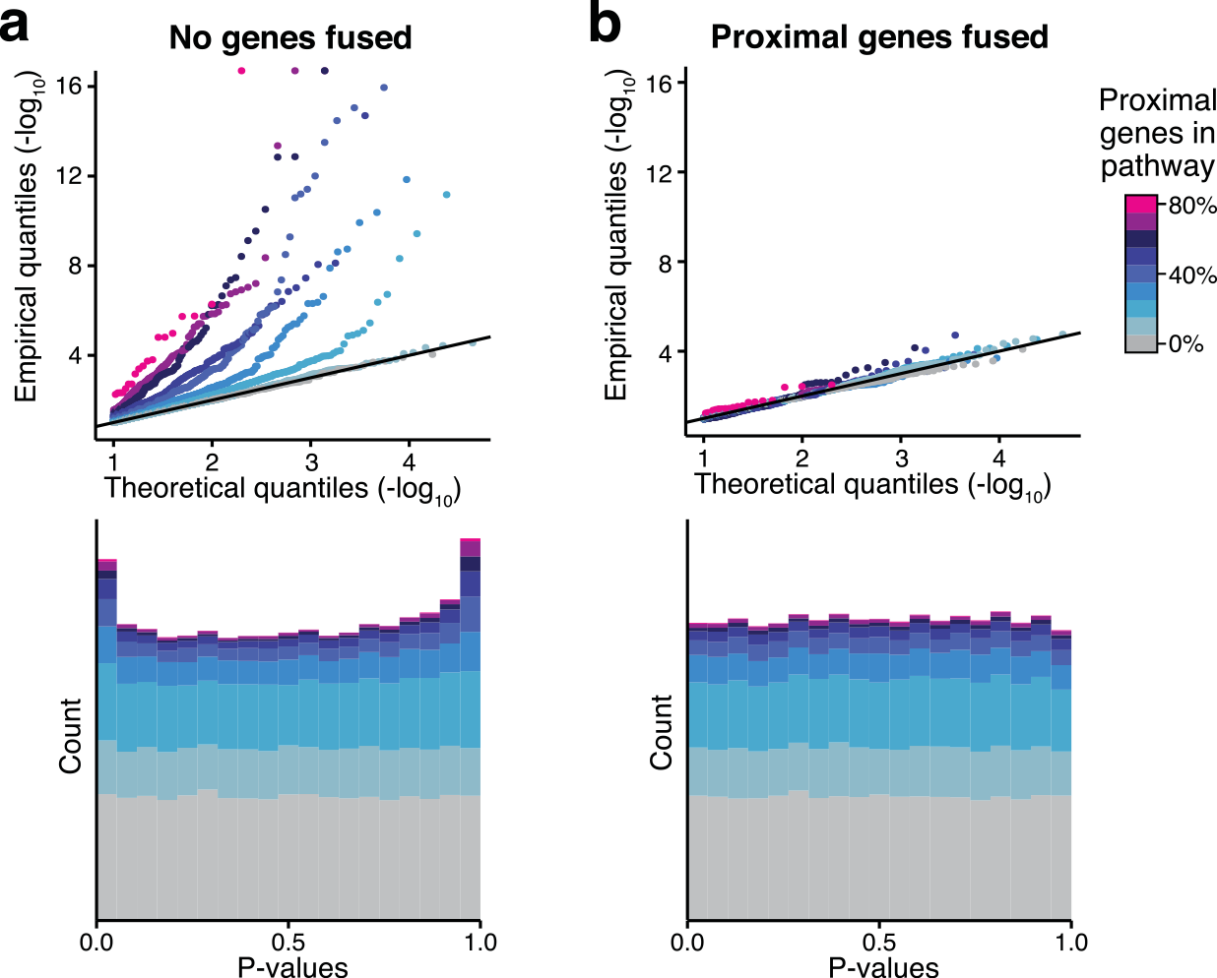
Pathway scores for random phenotypes. As input data we used 100 simulated instances of a random Gaussian phenotype and genotype data for 379 individuals from the EUR-1KG panel. Using the *Pascal* pipeline with sum gene scores and chi-squared pathway integration strategy we computed p-values for 1,077 pathways from our pathway library (results for max gene scores are similar, see S4 Fig). Panel (a) shows the p-value distributions without merging of neighbouring genes and (b) with merging of neighbouring genes (gene-fusion strategy).P-value distributions are represented by QQ-plots (upper panels) and histograms (lower panels). Results are colour-coded according to the fraction of genes in a given pathway that have a neighbouring gene in the same pathway, i.e. that are located nearby on the genome (distance <300kb). (a) P-values of pathways that contain genes in LD are strongly inflated without correction. (b) The gene fusion approach provides well-calibrated p-values independently of the number of pathway genes in LD.

To see if our approach provides well-calibrated p-values, we simulated random phenotypes and calculated association p-values for all 1KG SNPs. We then employed our pathway analysis pipeline and checked if pathway p-values were uniformly distributed, as expected for random phenotypes. We found that without the gene-fusion strategy, pathway p-values are indeed inflated and, as expected, this inflation is stronger for pathways with many proximal genes (Fig 3A). In contrast, applying the gene-fusion strategy corrects the distribution of pathway score p-values to be uniform irrespective of the number of proximal genes (Fig 3B). Importantly, we did not see inflation for very small p-values with the gene-fusion strategy, which is essential for type I error control.

Going one step further, we also simulated *in-silico* phenotypes influenced by randomly selected causal SNPs. We explored two scenarios: one where 50 SNPs were randomly selected from the entire genome and another where random sampling was applied to gene regions only. The experiment was repeated 50 times and independent genetic data was used to generate the estimated pairwise correlation. Although in this case gene scores naturally deviate from the null distribution, we found that overall pathway p-values remain well calibrated (S14 and S15 Figs). Note that we explored only a limited set of simulation scenarios and cannot exclude that some settings might produce less well-calibrated results (see legend of S15 Fig).

### *Pascal* has higher sensitivity and specificity than hypergeometric pathway enrichment tests

A commonly used statistic to derive pathway scores from a ranked list of genes (or SNPs) is to first apply a fixed threshold in order to define a subset of elements that is considered to be significantly associated with the given trait. The pathway statistic is then computed using a hypergeometric test evaluating whether the pathway contains more significant elements than expected. This approach is implemented, for example, in the tool MAGENTA[3]. Another common strategy is to use the rank-sum (Wilcoxon) test[3,28,29].

**Fig 4:**
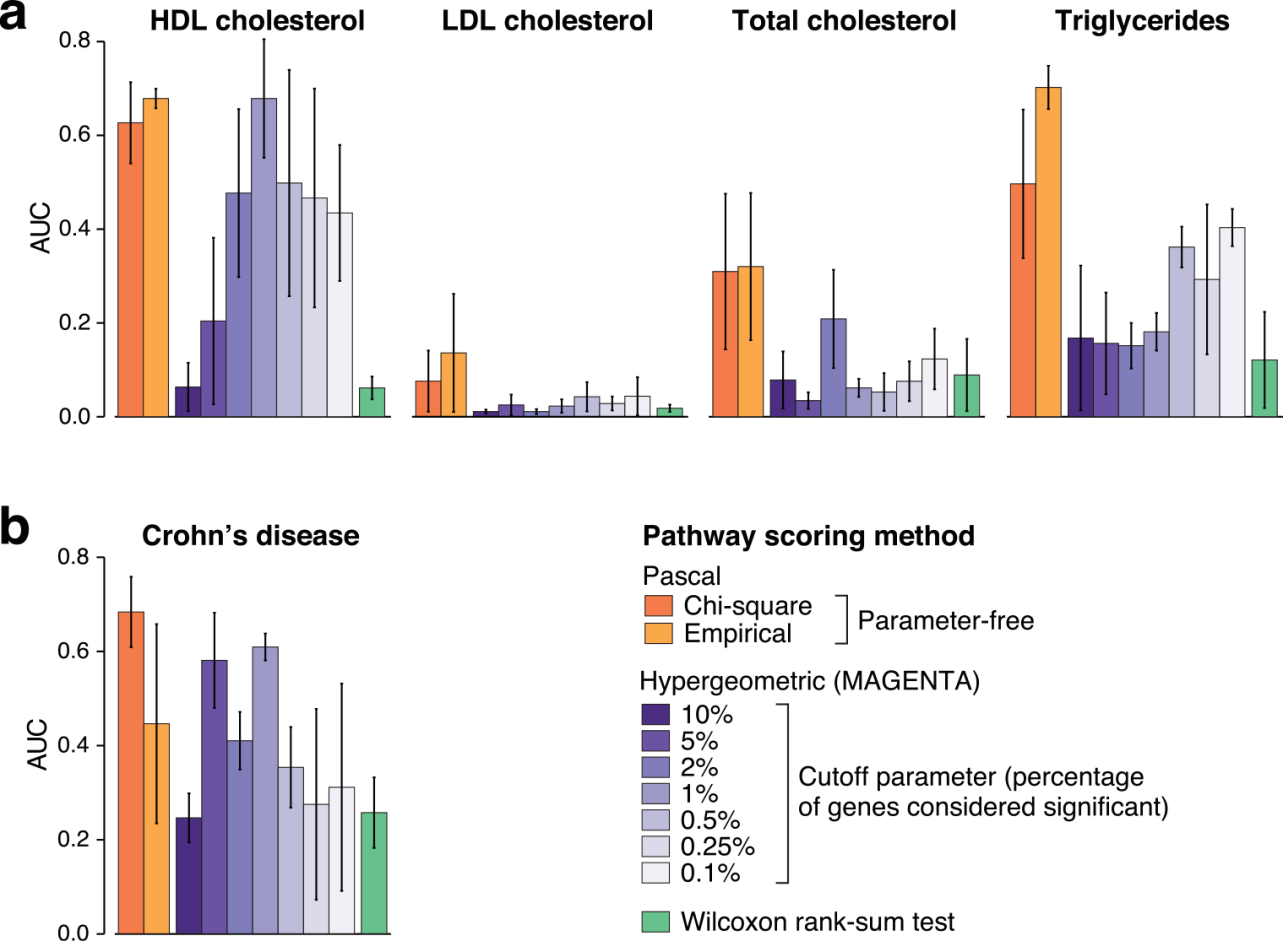
Performance of pathway enrichment methods for blood lipid traits and Crohn’s disease. Displayed is the mean area under the precision-recall curve (AUC) for pathways identified using *Pascal*, a standard hypergeometric test at various gene score threshold levels, and a rank-sum test (vertical bars show the standard error). We show results for the max gene scores (sum gene score results are similar, see S5 Fig). a) Results for four blood lipid traits. The gold standard pathway list was defined as all pathways that show a significance level below 5×10^−6^ for *any* of the tested threshold parameters for hypergeometric tests in the largest study of lipid traits to date[23]. The significance level of 5×10^−6^ corresponds to the Bonferroni corrected, genome-wide significance threshold at the 0.5% level for a single method. For each phenotype, error bars denote the standard error computed from three independent subsamples of the *CoLaus* study (including 1500 individuals each). We see good overall performance of *Pascal* pathway scores, whereas results for discrete gene sets vary widely with the particular choice for the threshold parameter of hypergeometric test. b) Results for Crohn’s disease using the same approach as in (a). A reference standard pathway list was defined as in (a) using the largest study of Crohn’s disease traits to date[31]. We observe that the chi-squared strategy performs at least as well as all other strategies in this setting, whereas performance of the hypergeometric testing strategy varies.

As described above, *Pascal* computes aggregate statistics without the need for defining a set of significant genes. We thus sought to compare this strategy with methods based on the hypergeometric or rank-sum tests. To this end, we tested performance on association results for four blood lipid traits obtained from of the *CoLaus* cohort[27]. We used a large meta-analysis of 188,577 individuals to define a reference set of associated pathways for each of the four lipid traits[23]. We then applied both pathway analysis methods to three non-overlapping, small subsets (1500 individuals) of the *CoLaus* study and compared how well the resulting pathways matched the reference set from the large study. We used the area under the precision-recall curve (AUC-PR) to quantify the performance of each method. Note that our choice was driven by the fact that precision-recall curves are preferred over receiver-operator-characteristic (ROC) curves when only a small fraction of tested pathways are in the reference set[30]. Our results show that *Pascal* outperforms both the hypergeometric and rank-sum based approaches (Fig 4A). Importantly, the better performance of *Pascal* is observed across a range of thresholds defining significant genes, including the optimal choice which is variable and *a priori* unknown across the different lipid phenotypes.

We applied the same evaluation strategy for GWAS data on Crohn’s disease. We used the currently largest GWAS for Crohn’s disease[31] to define a reference standard of associated pathways. We then applied both pathway analysis methods to results from two individual cohorts participating in the meta-analysis that contained at least 1000 cases[31–33]. We observed that the chi-squared-method performed at least as well as all other strategies in this setting (Fig 4B). Overall, we saw similar results for both max and sum gene scores (S5 Fig).

### *Pascal* has higher power than hypergeometric test based pathway enrichment in a wide range of traits

Having established that *Pascal* accurately controls type I error rate for simulated phenotypes and better recovers truly associated pathways for blood lipid traits as well as Crohn’s disease, we next sought to evaluate its power when applied to large meta-analytic studies on a broad range of traits, where no ground truth can be defined.

**Fig 5:**
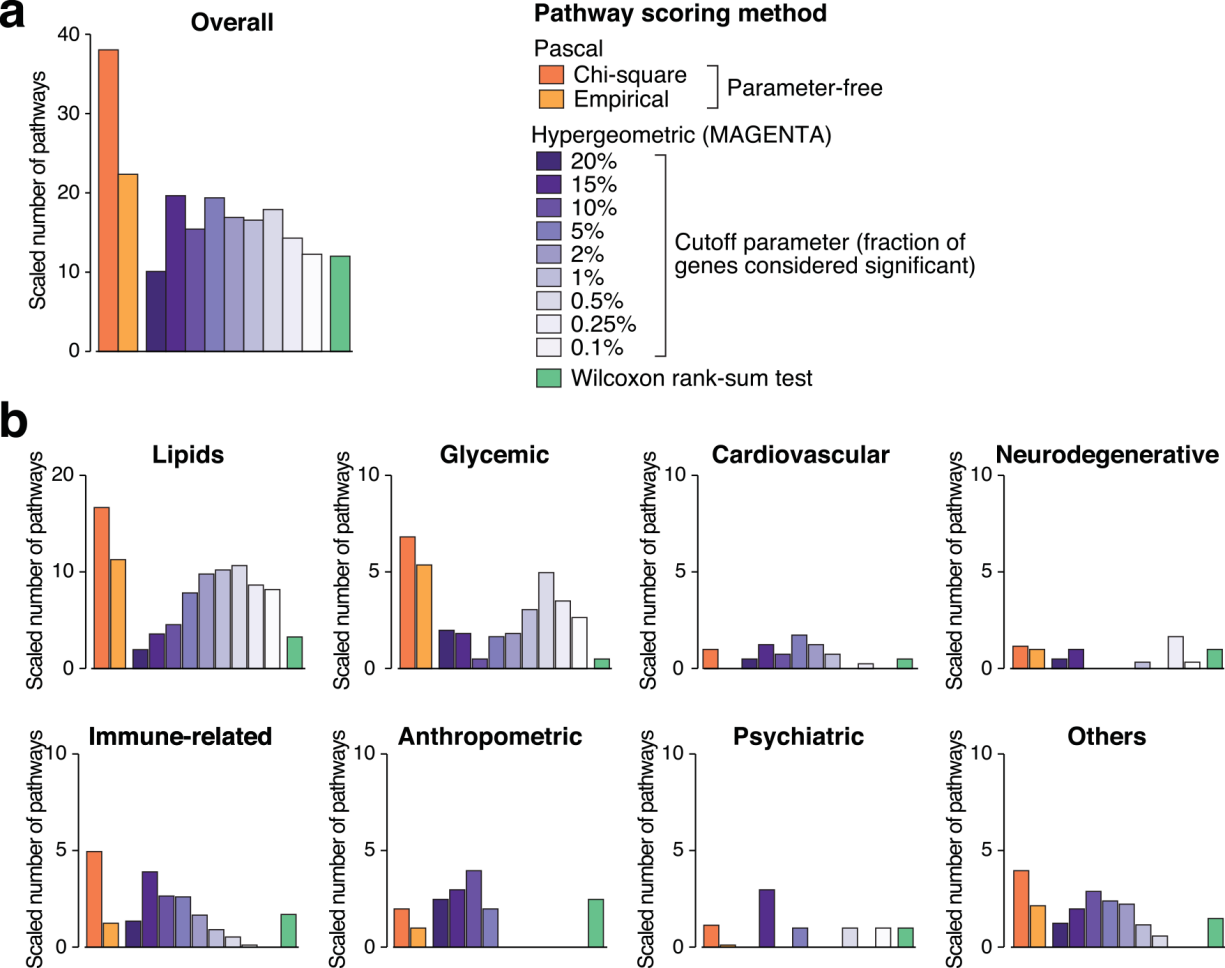
Power of pathway scoring methods across diverse traits and diseases. Bar heights represent the number of pathways found to be significant after Bonferroni-correction. Within a given trait group, results are aggregated for all tested GWAS studies. 65 GWAS had at least one significant pathway in one of the tested methods. For each GWAS, the raw number of significant pathways was divided by the number of pathways found by the best performing method. This was done in order to avoid that a few studies with many emerging pathways dominate. We show results for the MOCS gene scores (SOCS gene score results are similar, see S6 Fig). (a) Results are aggregated over all trait groups. (b) Results for different trait groups.

To this end, we compared *Pascal* with the methods based on the hypergeometric test (using 9 different threshold values) and the rank-sum test proposed by Segrè *et al*.[3] for 118 GWAS (S1 Table). All GWAS were derived from European populations justifying the use of the European 1KG genotypes as reference population. For a given GWAS, we asked how many tested pathways reached genome-wide significance at the Bonferroni-corrected p-value threshold of 0.05. Our results indicate that globally our approach has higher power than either the methods using the hypergeometric test (across all tested thresholds), or the rank-sum test (Figs 5A and S6). For individual traits (Fig 5B), specific choices of the threshold parameter of the hypergeometric test sometimes reveal more pathways, but again the value of the optimal threshold varies across traits and cannot be known *a priori*.

When splitting the GWAS into high powered (more than 50,000 individuals) and low powered studies (less than 50,000 individuals), we saw that in both cases we gain power by using *Pascal* although the effect was more pronounced for low powered GWAS (S7 Fig).

Hypergeometric enrichment testing is hampered by the fact that the optimal threshold is not known in advance. A strategy to overcome this could be to merge hypergeometric pathway scores coming from different sets of thresholds, further corrected for the effective size of the threshold sets. While such an aggregated hypergeometric testing improved performance, it was still outperformed by *Pascal* (S10 and S11 Figs).

One of the proposed pathway scoring methods transforms the ranked gene p-values such that they follow a chi-squared distribution. The chi-squared distribution is a special case of the Gamma distribution with shape parameter 0.5. Thus we also examined whether using other shape parameters of the Gamma distribution could improve performance (see Methods, S12 and S13 Figs). This analysis suggested that the chi-squared pathway scoring method represents a good compromise for a wide range of genetic architectures.

**Fig 6:**
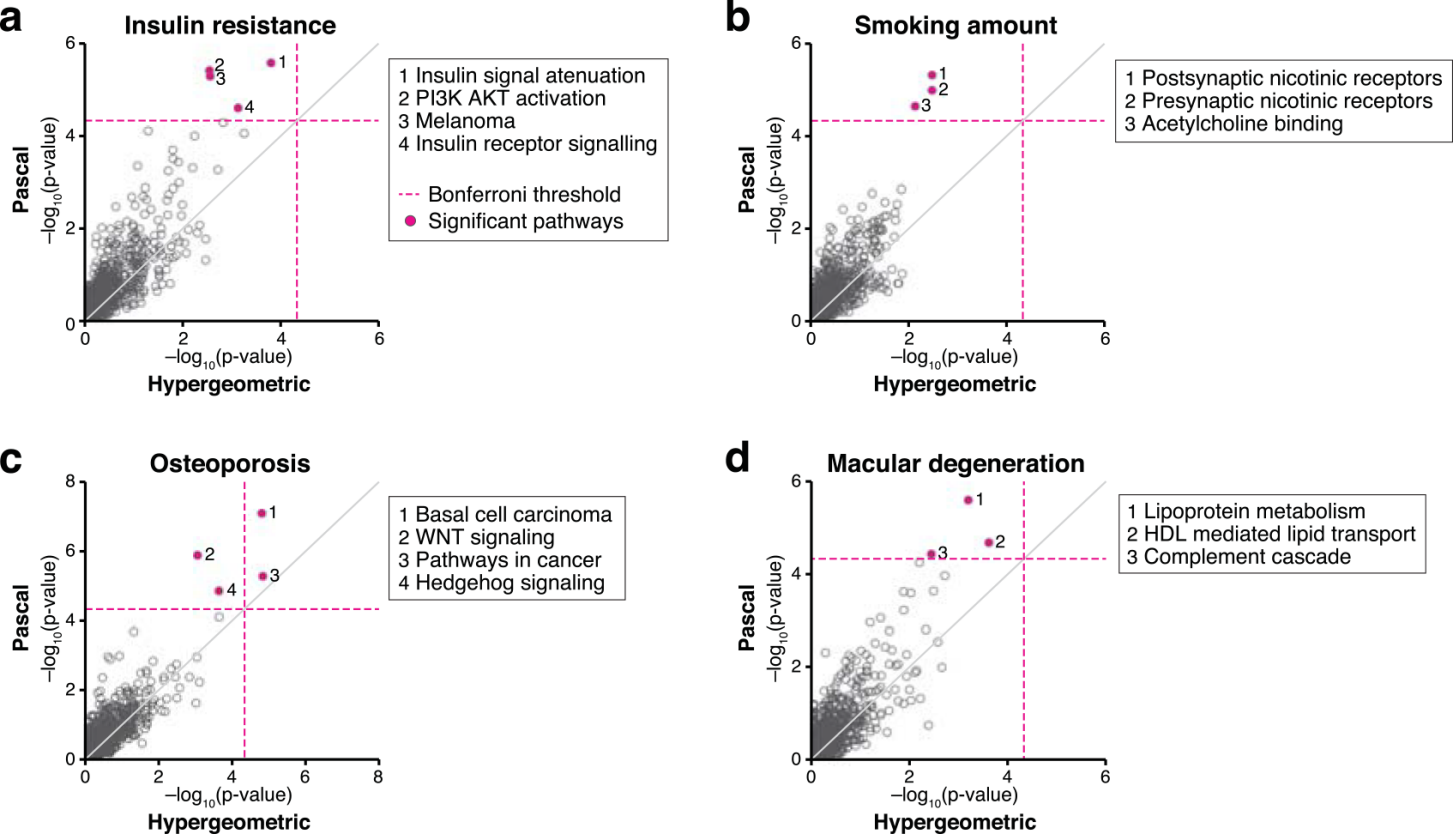
Examples of pathway enrichments comparing *Pascal* (chi-squared method) to the hypergeometric method. Displayed are results for four phenotypes showing improvement when using *Pascal* instead of the hypergeometric (binary) enrichment strategy at the 5% threshold level. Underlying gene scores were calculated using the sum method. Dashed lines refer to the Bonferroni significance level when correcting for the number of pathways (1077). Besides from few cancer-related pathways, all pathways highlighted by this analysis have been implied by prior research (see main text). (a) For the trait *insulin resistance*, Pascal scored the pathway *insulin signal attenuation* first, followed by two other trait-relevant pathways (*PI3K AKT activation* and *insulin receptor signaling*), while the hypergeometric test did not find any significant pathways. (b) For *smoking amount* (number of cigarettes per day), Pascal revealed three significant pathways related to *nicotinic acetylcholine receptors*. (c) For *osteoporosis*, two cancer-related pathways scored significant using both Pascal and the hypergeometric test, but only Pascal revealed the WNT and Hedgehog signaling pathways, which are known to be involved in osteoblast biology. (d) For *macular degeneration*, Pascal found three significant, trait-relevant pathways related to lipoproteins and the complement system.

We found numerous examples of biologically plausible pathways discovered by *Pascal* that were not found by a standard binary enrichment analysis (Fig 6, S2 Table). For insulin resistance[34] we found the REACTOME pathway *insulin signal attenuation* to be genome-wide significant. Notably, none of the genes in this pathway was found to contain a genome-wide significant SNP in the original publication. Another example is bone mineral density in women (LS-BMD)[35]. We found the *Hedgehog* and *Wnt* pathways to be significant, both of which are known to be involved in osteoblast biology[36]. Again, standard binary enrichment did not reach genome-wide significance. For smoking behaviour (measured in cigarettes per day)[37], we found pathways related to *nicotinic acetylcholine receptors*. For macular degeneration, we found *lipoprotein* and *complement system* involvement, which both have support in the literature[38,39]. These examples illustrate that the improvements made by *Pascal* not only lead to better performance on benchmarks, but may also have a dramatic impact on the interpretation of GWAS results in practice.

## Discussion

In this work, we presented a new tool called *Pascal* (*Pa*thway *sc*oring *al*gorithm) that specifically addresses both gene scoring and pathway enrichment, making significant advancement with respect to the state-of-the-art:

First, our gene score calculation combines analytical and numerical solutions to properly correct for multiple testing on correlated data[21]. While some of these approaches have already been applied within the rare variant field[20] (typically in a gene-wise fashion) we provide a streamlined implementation that can run genome-wide analyses without the need for any Monte Carlo simulations (making it about 100 times faster and more precise than the widely used software VEGAS).

Second, our pathway scoring integrates individual gene scores without the need for a tuneable threshold parameter to dichotomize gene scores for binary membership enrichment analysis (as done for example by MAGENTA). The choice of such a parameter is not straightforward and our method usually performs better, regardless of the chosen parameter.

Third, we show that the null distribution of enrichment p-values for pathways that contain genes in linkage disequilibrium is non-uniform due to an “over-counting” of gene association signals. This is a potential source of type I error underestimation and our method corrects for this phenomenon using a *gene fusion* approach, which considers genes in LD as single entities.

We have extensively evaluated the performance of *Pascal* for several real data sets. These comparisons demonstrated the rigorous control of type I error and superior predictive power in a wide range of trait and power settings in terms of enhanced precision-recall curves.

As an additional global measure of power, we considered the number of significantly enriched pathways for a large number of GWAS meta-analysis summary statistics. On average, our approach resulted in higher numbers of significant pathway scores than any binary enrichment strategy. Given its precise type I error control, this provides additional evidence of increased power for a wide range of traits. Indeed, the elevated rate of putatively involved pathways produced by our method not only reflects its higher sensitivity, but also already generates new hypotheses for further studies.

Taken together, our results demonstrate the superior performance of our approach compared to standard binary enrichment and rank-sum tests. Although methods with tuneable parameters might yield improved results in a particular setting, it is difficult to predict the optimal parameter choice. Indeed, the optimal parameter depends on sample size, as well as complexity and heritability of the phenotype. Another issue with binary enrichment tests is that the hypergeometric distribution is discrete, which leads to conservative p-values, especially if the expected number of successful draws is low. Our pathway scoring approach avoids this problem. Also, our approach lends itself to naturally extending pathway scoring in case genes have probabilistic membership in predefined pathways.

Users of our method will still have to make two choices: how to convert SNP p-values to gene scores (max or sum gene scores), and how to transform gene scores into pathway scores (empirical or chi-squared). We do not see evidence that one gene scoring method systematically outperforms the other in the context of our chi-squared pathway scoring method, while there seems to be a better performance for sum gene score when using the empirical approach (S8 Fig). To investigate this phenomenon we winsorized p-values (i.e. extreme p-values below 10^−12^ were set to 10^−12^) and saw that the max gene score combined with empirical sampling suffered far less performance loss (S9 Fig). We therefore conclude that the power loss is due to outlier gene scores. The max gene-scores can lead to very high gene scores for high-powered studies. In the extreme case one gene might reach scores so high that it precludes detection of pathways not containing that gene when *the empirical sampling* strategy is used.

Future work could attempt to enhance several other aspects of our pathway enrichment analysis. For example, here we mapped SNPs to genes only based on physical distance, while potential improvements could be attained by incorporating additional information, such as eQTL data[40] and functional annotations, to assign weights to different association signals within a locus. While our approach is amenable to such a weighting scheme, this would potentially require the introduction of tuneable parameters, which we avoided so far. Furthermore, one may attempt to redefine gene sets based on external unbiased large-scale molecular data, such as expression data, while so far we only used the established (but likely biased) pathway collections[4]. To this end, we already integrated *Pascal* into a pipeline to analyze the connectivity between trait-associated genes across over 400 tissue-specific regulatory, co-expression and protein-protein interaction networks, further demonstrating its value for network-based analysis of GWAS results (Marbach *et al*., submitted).

As an additional caveat, we should mention that *Pascal* uses the European 1KG sample as reference population per default. This choice may not be appropriate if the studied sample is not of European origin. In this case the user is encouraged to supply *Pascal* with the appropriate reference panel. Also, SNPs with low MAF are by default excluded from the analysis, because the low number of individuals in the European 1KG sample limits the accuracy of the LD estimate for low frequency variants. If the user wishes to include lower frequency variants, the use of a reference sample containing more individuals is recommended.

To conclude, *Pascal* implements fast and rigorous analytical methods into a single analysis pipeline tailored for gene scoring and pathway enrichment analysis that can be run on a desktop computer. We thus hope that *Pascal* will be useful to the GWAS community in a range of applications and play a pivotal role in leveraging the rich information encoded in GWAS results both for single traits and—given its efficiency and power—in particular also for high-dimensional molecular traits.

Our tool is available as a single standalone executable java package containing all required additional data at: http://www2.unil.ch/cbg/index.php?title=Pascal (short URL: http://goo.gl/t4U5z6).

## Materials and Methods

### Gene scores

The *Pascal* gene scoring method consists of the following steps (Fig 1A). First, we assign SNPs to genes if they are located within a given window around the gene body. For the experiments reported in this paper, we used windows extending 50kb up and downstream from the gene. A reference population is required to estimate the correlation structure between Z-scores of SNP association values. Here, we used the European population of the 1000 Genomes Project (1KG)[22], which allows us to apply our approach flexibly to summary statistics from diverse panels (HapMap, 1KG imputed, metaboChip or ImmunoChip).

Under the null hypothesis, it can be shown that the Z-scores of *n* SNPs in our gene region as multivariate normal:

$$z∼N_{n}\left(0,\Sigma \right)$$

where **Σ** is the pair-wise SNP-by-SNP correlation matrix (see Section ‘Derivation of the sum score’ for details).

We define our base statistics, the SOCS (*T*_*sum*_) and MOCS (*T*_*max*_), as:

$$T_{sum}\;=\sum_{i=1}^{n}\;z_{i}^{2}$$

and

$$T_{max}\;=\text{max}\left(z_{i}^{2}\right),$$

respectively. It can be shown that *T*_*sum*_ is distributed according to the weighted sum of $\chi_{1}^{2}$-distributed random variables:

$$T_{sum}∼\sum_{i}\lambda_{i}\chi_{1}^{2}$$

where *λ*_*i*_ is the *i*-th eigenvalue of **Σ**. Its distribution function can be evaluated numerically (see Section Algorithmic details for gene-score calculations for details). To estimate the null distribution of *T*_*max*_ we make use of the fact that

$$\text{P}\left[T_{max}\geq t\right]=\text{P}\left[max\left(\left|z_{i}\right|\right)\geq t\right]=1-P(|z_{i}|<t,i=1,2,\ldots \;n).$$

This amounts to a rectangular integration over a multivariate normal, for which an efficient algorithm is available[41]. The current implementation of this integration is suitable to estimate p-values larger than 10^−15^. To approximate gene-wise p-values below this limit we multiply the minimum p-value of SNPs in the region with the effective number of tests within the gene (see Section Algorithmic details for gene-score calculation).

### Gene fusion

Pathway analysis methods typically assume that the gene scores used to define pathway enrichment are independent. However, functionally related genes often cluster on the genome and harbor SNPs in LD, leading to correlated gene scores that violate this assumption. To circumvent this problem, we check for a given pathway if any of its genes that cluster physically close on the chromosome are in LD. If so, for the calculation of the pathway score, we consider a single entity (a so-called *fusion-gene)* consisting of all the SNPs of the gene cluster. We then replace the genes in the cluster by this *fusion-gene* and calculate its gene score, but only for the calculation of the score for this particular pathway. The pathway score is then computed from the p-values of independent pathway genes and fusion genes that integrate the associational signals from dependent pathway genes (Fig 1B). In this way, the LD structure of neighbouring pathway genes is taken into account. Our gene scoring method facilitates this approach because it is sufficiently fast and scalable for recomputing the scores of all fusion genes.

### Pathway scores

For pathway analysis, we propose a parameter free enrichment strategy that does not require the specification of a gene score threshold, and thus allows weakly associated genes to contribute to pathway enrichment. The general approach consists of three steps: (1) gene scores are transformed so that they follow a target distribution, (2) a test statistic is computed by summing the transformed scores of pathway member genes and fusion-genes, and (3) analytic or empirical methods are used to evaluate whether the observed test statistic is higher than expected, i.e., the pathway is enriched for trait-associated genes. We considered two variants of this approach for pathway scoring (see overview in S1 Fig). The first variant is termed as the ***chi-squared method***:

1. Gene score p-values are ranked such that the lowest p-value gets the highest rank. The rank value is then divided by the number of genes plus one to obtain a uniform distribution.
2. Uniform distribution values are transformed by the $\chi_{1}^{2}$-quantile function to obtain a $\chi_{1}^{2}$-distribution of gene scores.
3. $\chi_{1}^{2}$–gene scores of a given pathway of size *m* are summed and tested against a $\chi_{m}^{2}$-distribution.

The second variant is the ***empirical sampling*** method:

1. Gene score p-values are directly transformed with the $\chi_{1}^{2}$-quantile function to obtain new gene scores: $F_{\chi_{1}^{2}}^{-1}\left(1-p\right)$.
2. A raw pathway score for a pathway of size *m* is computed by summing the transformed gene scores for all pathway genes.
3. A Monte Carlo estimate of the p-value is obtained by sampling random gene sets of size *m* and calculating the fraction of sets reaching a higher score than gene set of the given pathway.

We also tested a generalization of the chi-squared method where the inverse $\chi_{1}^{2}$-quantile transformation of the p-value ranks was replaced by the inverse Gamma-quantile transformation with varying shape parameter. For shape parameter of 0.5, the results coincide with results from the chi-squared method.

For our benchmarking procedures we created a pathway library by combining the results from KEGG[7,42], REACTOME[9] and BIOCARTA[10] that we downloaded from MsigDB[43].

### Derivation of the sum-score

Let **z** be the vector of Z-statistics coming from regressing the phenotype on each of the *n* SNPs within a gene-region. By construction, each Z-statistic has zero mean under the null. When both the outcome trait and the genotypes are standardized, the linear regression Z-statistics are essentially the scalar products of the genotype and the phenotype vectors. In other words, each Z-statistic in the region represents a weighted average of the same set of independent, identically distributed random variables. It can be shown that the correlation between two such mixtures, i.e. two Z-statistics, equals to the correlation between the weights, i.e. the correlation between the corresponding SNPs. Thus, the covariance matrix of **z** is simply the pairwise SNP-by-SNP correlation matrix, denoted by **Σ**. Furthermore, the central limit theorem ensures that in case of sufficiently large sample size the Z-statistics are normally distributed. These facts put together yield that–under the null-hypothesis that no signal is present–**z** follows a multivariate normal distribution, $z∼N_{n}\left(0,\Sigma \right)$. For a detailed derivation see supplementary material in Xu et al[44] for example. Note that the between SNP correlation matrix **Σ** can be estimated from external data[17,45].

The eigenvalue decomposition of **Σ** is

$$\Sigma =\Gamma \Lambda \Gamma^{T},$$

where **Γ** and **Λ** are the matrices of eigenvectors and eigenvalues, respectively. We see that multiplying ***z*** with the inverse of the square-root of ***Σ*** leads to a vector of independent random variables. Let **y** be defined as

$$y=\Lambda^{-1/2}\Gamma^{T}z,$$

then

$$y∼N_{n}\left(0,I_{n}\right).$$

It follows that

$$z^{T}z=z^{T}\Gamma \Gamma^{T}z=y^{T}\Lambda y∼\sum_{i}\;\lambda_{i}\chi_{1}^{2},$$

where *λ*_*i*_ is the *i*-th eigenvalue of **Σ** and $\chi_{1}^{2}$ represents the chi-squared distribution.

### Parameter settings

If not stated otherwise, our tool was always used with the following settings. We extended gene regions by 50kb upstream and downstream for gene scoring. Only SNPs that reached a MAF of 0.05 in European 1KG sample were used. For pathway score calculation, we removed the HLA-region. The gene-fusion parameter was set to 1Mb, so that when calculating a particular pathway score, all pathway-member genes less than 1Mb apart were fused for the calculation. We also removed genes containing more than 3000 SNPs except during speed benchmarking (Fig 2) where all SNPs were used.

### Simulation settings for type I error control of the pathway scores

We used genotypes for 379 individuals from the EUR-1KG cohort[22]. Corresponding phenotype values were simulated as independent, standard normally distributed variables. Univariate Z-scores for each of the 2,692,429 tested SNPs were calculated using linear regression. Simulations were repeated 100 times. Since we investigated the impact of gene-fusion, the LD matrix was estimated from the same data set to avoid any influence that might come from out-of-sample LD estimation.

### Algorithmic details for gene-score calculations

#### Max-score

The algorithm first tries to use Monte Carlo simulation to derive p-values. Should the p-value be too small to be estimated within a few Monte Carlo draws, the procedure makes use of an algorithm for rectangular multivariate normal integration[41]. The implementation of the integration algorithm that is used is suitable to estimate p-values larger than 10^−15^. In addition, this implementation is limited to correlation matrices of size below 1000 due to numerical stability concerns. Therefore, SNPs that are in very high LD (r^2^ > 0.98) are pruned to lower the size of the correlation matrix. If more than 1000 SNPs fall into the gene or the gene-wise p-value is below 10^−15^, we approximate the gene score by multiplying the minimal SNP-wise p-value in the gene region by the effective number of tests. The effective number of tests is calculated as the minimum number of principal components needed to explain 99.5% of total variance[46].

#### Sum-score

The algorithm relies on the Davies algorithm to calculate distribution function values of weighted sums of independent $\chi_{1}^{2}$-distributed random variables[47]. In case of convergence problems the Farebrother algorithm is used as a backup[48,49].

#### Web resources

A stand-alone executable for *Pascal* can be found at http://www2.unil.ch/cbg/index.php?title=Pascal. The *Pascal* source code can be found at https://github.com/dlampart/Pascal.

## Supporting Information

S1 FigOverview of pathway scoring strategies in *Pascal*.Pathway scores are computed from gene scores. The upper panel shows a typical gene score distribution, where the pathway gene scores are indicated in black. In order to compute pathway scores, the original gene score p-values need to be transformed. To this end we use one of two strategies: in our *empirical strategy* (lower left panel), gene score p-values are directly transformed with the inverse $\chi_{1}^{2}$-quantile function $F_{\chi_{1}^{2}}^{-1}\left(1-p\right)$ to obtain scores, which are then summed across all pathway genes. A Monte Carlo estimate of the p-value is then obtained by sampling random gene sets of the same size and calculating the fraction of sets reaching a higher score than that of the given pathway. In the *chi-squared method* (bottom right panel), the gene score p-values are first ranked such that the lowest p-value ranks highest. The rank values are then divided by the number of genes plus one to define new p-values (p_rank_) that are distributed uniformly by definition. From there, we proceed as for the empirical strategy just replacing p by p_rank_. Also, since the scores are guaranteed to be chi-squared distributed, the computation of their corresponding p-value can be done analytically without any loss in precision.(PDF)

S2 Fig Comparison of results for different reference panels. Comparing p-values computed using LD matrices from the European 1000 Genome reference panel and the *CoLaus* cohort. GWAS summary statistics were taken from a large-scale blood-HDL level meta-analysis. Results are compared for (a) max gene scores; (b) max gene scores excluding gene scores that were computed with the effective number of tests approximation; and (c) sum gene scores. There is good concordance in all cases.
(PDF)

S3 Fig Comparison of max and sum gene scores. We compared max and sum gene scores directly for a large-scale blood HDL level meta-analysis. Only gene scores up to 10^−15^ are displayed, which truncated 6 genes with very large max scores. R^2^ between the–log10-transformed variables is 90%. Max scores tend to be larger when the two methods do not agree.
(PDF)

S4 Fig Pathway scores for random phenotypes using max gene scores. P-values for 1077 pathways from our pathway library were computed for 100 random phenotypes using the *Pascal* pipeline using max gene scores and *chi-squared* pathway integration strategy (a) without merging of neighbouring genes and (b) with merging of neighbouring genes (gene-fusion strategy). P-value distributions are represented by QQ-plots (upper panels) and histograms (lower panels). Results are colour-coded according to the fraction of genes in a given pathway that have a neighbouring gene in the same pathway, i.e. that are located nearby on the genome (distance <300kb). (a) P-values of pathways that contain genes in LD are strongly inflated without correction. (b) The gene fusion approach provides well-calibrated p-values independently of the number of pathway genes in LD.
(PDF)

S5 Fig Performance of pathway enrichment methods for blood lipid traits and Crohn’s disease using sum of squares (SOCS) statistics for defining gene scores. Displayed is the mean area under the precision-recall curve (AUC) for pathways identified using *Pascal*, a standard hypergeometric test at various gene score thresholds, and a rank-sum test (vertical bars show the standard error). We show results for the SOCS gene scores (MOCS gene score results are similar, see Fig 4 in the main text). a) Results for four blood lipid traits. A reference standard pathway list was defined as all pathways that show a significance level below 5×10^−6^, for *any* of the tested threshold parameters for hypergeometric tests in the largest study of lipid traits to date. The significance level of 5×10^−6^ corresponds to the Bonferroni corrected, genome-wide significance threshold at the 0.5% level for a single method. For each phenotype, error bars denote the standard error computed from three independent subsamples of the *CoLaus* study (including 1500 individuals each). We see good overall performance of *Pascal* pathway scores, whereas results for discrete gene sets vary widely with the particular choice for the threshold parameter of hypergeometric test. b) Results for Crohn’s disease using the same approach as in (a). A reference standard pathway list was defined as all pathways that show a significance level below 5×10^−6^ for *any* of the tested threshold parameters for hypergeometric tests in the largest study of Crohn’s disease traits to date. We observe that the chi-squared strategy outperforms all other strategies in this setting, whereas performance of the hypergeometric testing strategy varies.
(PDF)

S6 Fig Power of pathway scoring methods across diverse traits and diseases using sum of squares (SOCS) statistics for defining gene scores. Bar heights represent the number of pathways found to be significant after Bonferroni correction. Within a given trait group, results are aggregated for all tested GWAS studies. 65 GWAS had at least one significant pathway in one of the tested method. For each GWAS, the raw number of significant pathways was divided by the number of pathways found by the best performing method. This was done to avoid that a few studies with many emerging pathways dominate. We show results for the SOCS gene scores (MOCS gene score results are similar, see Fig 5). (a) Results are aggregated over all trait groups. (b) Results for different trait groups.
(PDF)

S7 Fig Power of pathway scoring methods stratified with respect to sample size. Only GWAS studies for quantitative traits were used. Top panels (a,b) show results for max gene scores and bottom panels (c,d) show results for sum gene scores. (a,c) Results for all studies where the number of individuals was below 50,000. (b,d) Results for studies with sample sizes above 50,000. We see power gains in all cases. The improvements are particularly pronounced in lower powered GWAS.
(PDF)

S8 Fig Power comparison max and sum gene scores for pathway analysis. Bar heights represent the number of pathways found to be significant after Bonferroni correction. Within a given trait group, results are aggregated for all tested GWAS studies. For each GWAS, the raw number of significant pathways was divided by the number of pathways found by the best performing method. Results for SOCS and MOCS as well as the chi-square and empirical pathway scores are displayed. We observe a drop in performance for the combination of MOCS gene scores with empirical pathway scores.
(PDF)

S9 Fig Power analysis for max gene scores with capped gene scores. Bar heights represent the number of pathways found to be significant after Bonferroni correction. Within a given trait group, results are aggregated for all tested GWAS studies. For each GWAS, the raw number of significant pathways was divided by the number of pathways found by the best performing method. Max gene scores using empirical sampling pathway scores (emp) and chi-squared pathway scores (chi2) are compared to max gene scores combined with empirical sampling, where outlier gene scores (p-value <10^−12^) are set to 10^−12^ (empCapped). We chose the capping value such that the maximum–log10 p-value was roughly in the middle between genome wide significance threshold (8) and the maximum value that can be calculated for the sum statistic (15).
(PDF)

S10 Fig Power of *Pascal* pathway scoring methods compared to aggregated hypergeometric scores (MOCS). The same data as in Fig 5 is plotted here. However, instead of comparing *Pascal* pathway scoring methods with results for all hypergeometric threshold separately, we defined a new aggregated pathway score that picks the optimal threshold for each pathway over a range of hypergeometric threshold and correcting for the multiple number of tests by Bonferroni correction. Results for different sets of thresholds are displayed. Set1 refers to the complete set of thresholds (i.e.: 25%, 15%, 10%, 5%, 2%, 1%, 0.25%, 0.1%). Set2 refers to a set with thresholds more ‘spread out’ (i.e.: 25%, 5%, 1%, 0.25). We see that *Pascal* has better performance, except when combining the ‘empirical sampling’ pathway scoring method with max gene scores.
(PDF)

S11 Fig Power of *Pascal* pathway scoring methods compared to aggregated hypergeometric scores (SOCS). The same data as in Fig 5 is plotted here. However, instead of comparing *Pascal* pathway scoring methods with results for all hypergeometric threshold separately, we defined a new aggregated pathway score that picks the optimal threshold for each pathway over a range of hypergeometric threshold and correcting for the multiple number of tests by Bonferroni correction. Results for different sets of thresholds are displayed. Set1 refers to the complete set of thresholds (i.e.: 25%, 15%, 10%, 5%, 2%, 1%, 0.25%, 0.1%). Set2 refers to a set with thresholds more ‘spread out’ (i.e.: 25%, 5%, 1%, 0.25). We see that *Pascal* has better performance.
(PDF)

S12 Fig Power of gamma distribution for pathway analysis (MOCS). Bar heights represent the number of pathways found to be significant after Bonferroni correction. Different bars signify results for a different gamma shape parameter value. For each GWAS, the raw number of significant pathways was divided by the number of pathways found by the best performing method. Upper left panel ‘All’ refers to all traits stacked. We present here MOCS gene score based results. 52 GWAS showed at least one significant pathway in one of the evaluated scenarios.
(PDF)

S13 Fig Power of gamma distribution for pathway analysis (SOCS). Bar heights represent the number of pathways found to be significant after Bonferroni correction. Different bars signify results for a different gamma shape parameter value. For each GWAS, the raw number of significant pathways was divided by the number of pathways found by the best performing method. Upper left panel ‘All’ refers to all traits stacked. We present here MOCS gene score based results. 60 GWAS showed at least one significant pathway in one of the evaluated scenarios.
(PDF)

S14 Fig Distribution of pathway scores for simulated phenotypes influenced by causal SNPs in coding regions. We first sampled 50 random SNPs assayed in *CoLaus* in or close to coding regions. Using the genotypes of the *CoLaus* study we then simulated phenotypes by adding up the sampled 50 SNPs with a normally distributed effect size with a variance of 0.04 plus Gaussian noise (with a variance of 1). We then ran GWAS for the simulated phenotype to obtain association summary statistics. The experiment was repeated 50 times. On average, this resulted in 18 independent, genome-wide significant gene score hits for each simulated GWAS (for the MOCS statistic). We applied *Pascal* to compute pathway scores for each of the 50 simulated GWAS. We found that the resulting pathway scores are well calibrated, i.e., they do not show inflation or deflation regardless of the setting used (max or sum gene score, chi2 or empirical enrichment test). The QQ-plots show the median value for each quantile across the 50 simulated GWAS. The shaded areas correspond to 95% confidence intervals for the median (estimated from 2000 bootstrap samples of size 50, with replacements). Similar results were obtained when varying the type and number of simulated causal SNPs and their effect size.
(PDF)

S15 Fig Distribution of pathway scores for simulated phenotype influenced by causal SNPs in coding and non-coding regions. These QQ-plots correspond to an analysis equivalent to that of S14 Fig but with 50 SNPs chosen uniformly from all SNPs assayed in *CoLaus*, rather than from genic regions only. On average, this resulted in 12 independent, genome-wide significant gene score hits for each simulated GWAS (using the MOCS statistic). Note that this does not completely exclude the possibility of less well-calibrated scores in other settings. Deviations from perfectly calibrated scores may occur in the cases where true SNP associations are present, because the gene wise test statistic may have varying power for different genes depending on the genetic architecture of the associated phenotype and on certain gene properties (such as gene length, LD structure, SNP coverage, or SNP allele frequency). If a set of pathways contains many pathways enriched (or depleted) for genes with such confounding factors, inflation or deflation is possible.
(PDF)

S1 Table Details of GWAS used. Details of the 118 GWAS that we used for comparing *Pascal* with other methods.
(TXT)

S2 Table Pathways found by *Pascal*. Tables of pathways discovered by *Pascal* for the 118 GWAS.
(TXT)
