# Supplementary figures and images for: Fast and Rigorous Computation of Gene and Pathway Scores from SNP-Based Summary Statistics
Source: PLoS Comput Biol. 2016 Jan 25;12(1):e1004714. doi: 10.1371/journal.pcbi.1004714 (PMC4726509; doi:10.1371/journal.pcbi.1004714)

original p-values

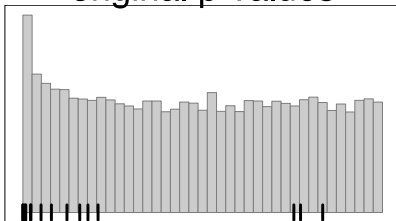

● Pathway  
Genes

$$F_{\chi_1^2}^{-1}(1 - p)$$

$$F_{\chi_1^2}^{-1}(1 - p_{rank})$$

empirical-strategy

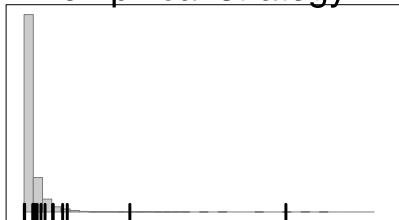

chi2-strategy

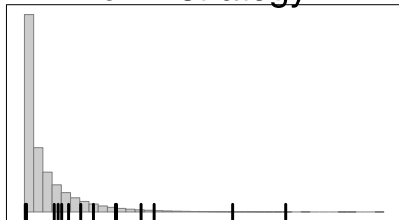

Supplement: S1 Fig — Pathway scores are computed from gene scores. The upper panel shows a typical gene score distribution, where the pathway gene scores are indicated in black. In order to compute pathway scores, the original gene score p-values need to be transformed. To this end we use one of two strategies: in our empirical strategy (lower left panel), gene score p-values are directly transformed with the inverse χ12-quantile function Fχ12−1(1−p) to obtain scores, which are then summed across all pathway genes. A Monte Carlo estimate of the p-value is then obtained by sampling random gene sets of the same size and calculating the fraction of sets reaching a higher score than that of the given pathway. In the chi-squared method (bottom right panel), the gene score p-values are first ranked such that the lowest p-value ranks highest. The rank values are then divided by the number of genes plus one to define new p-values (prank) that are distributed uniformly by definition. From there, we proceed as for the empirical strategy just replacing p by prank. Also, since the scores are guaranteed to be chi-squared distributed, the computation of their corresponding p-value can be done analytically without any loss in precision. (PDF) [file pcbi.1004714.s001.pdf]

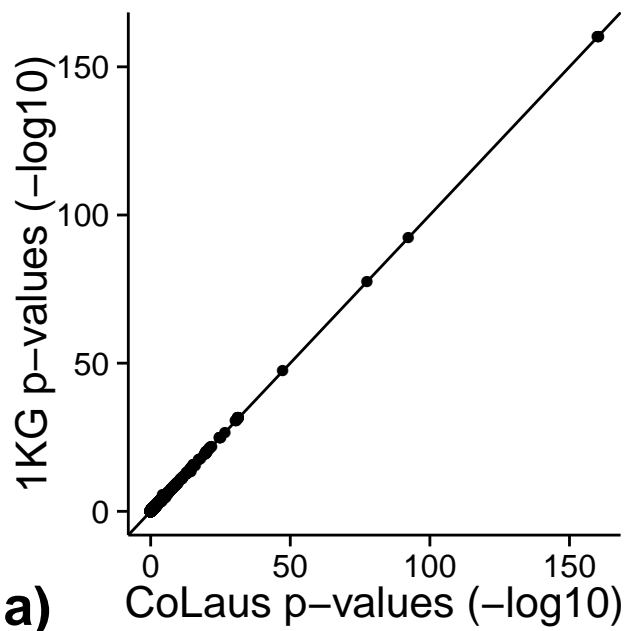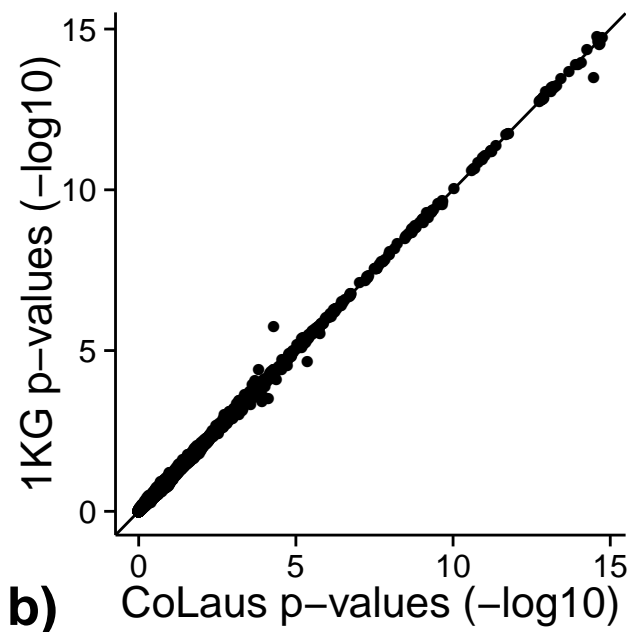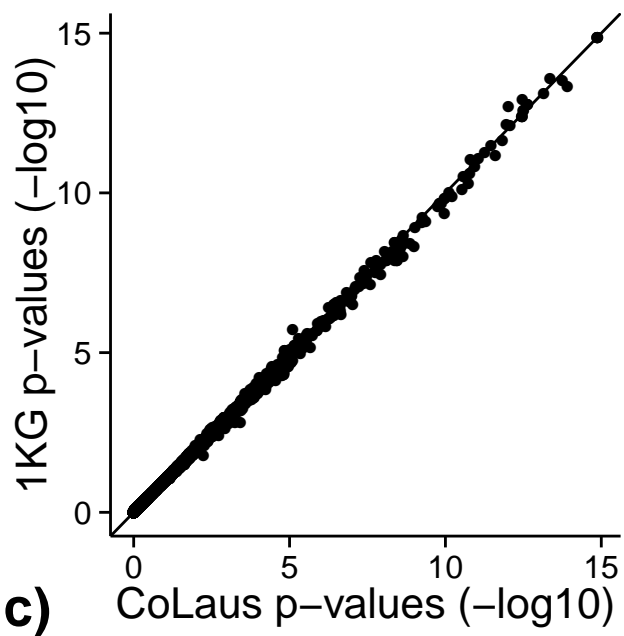

Supplement: S2 Fig — Comparing p-values computed using LD matrices from the European 1000 Genome reference panel and the CoLaus cohort. GWAS summary statistics were taken from a large-scale blood-HDL level meta-analysis. Results are compared for (a) max gene scores; (b) max gene scores excluding gene scores that were computed with the effective number of tests approximation; and (c) sum gene scores. There is good concordance in all cases. (PDF) [file pcbi.1004714.s002.pdf]

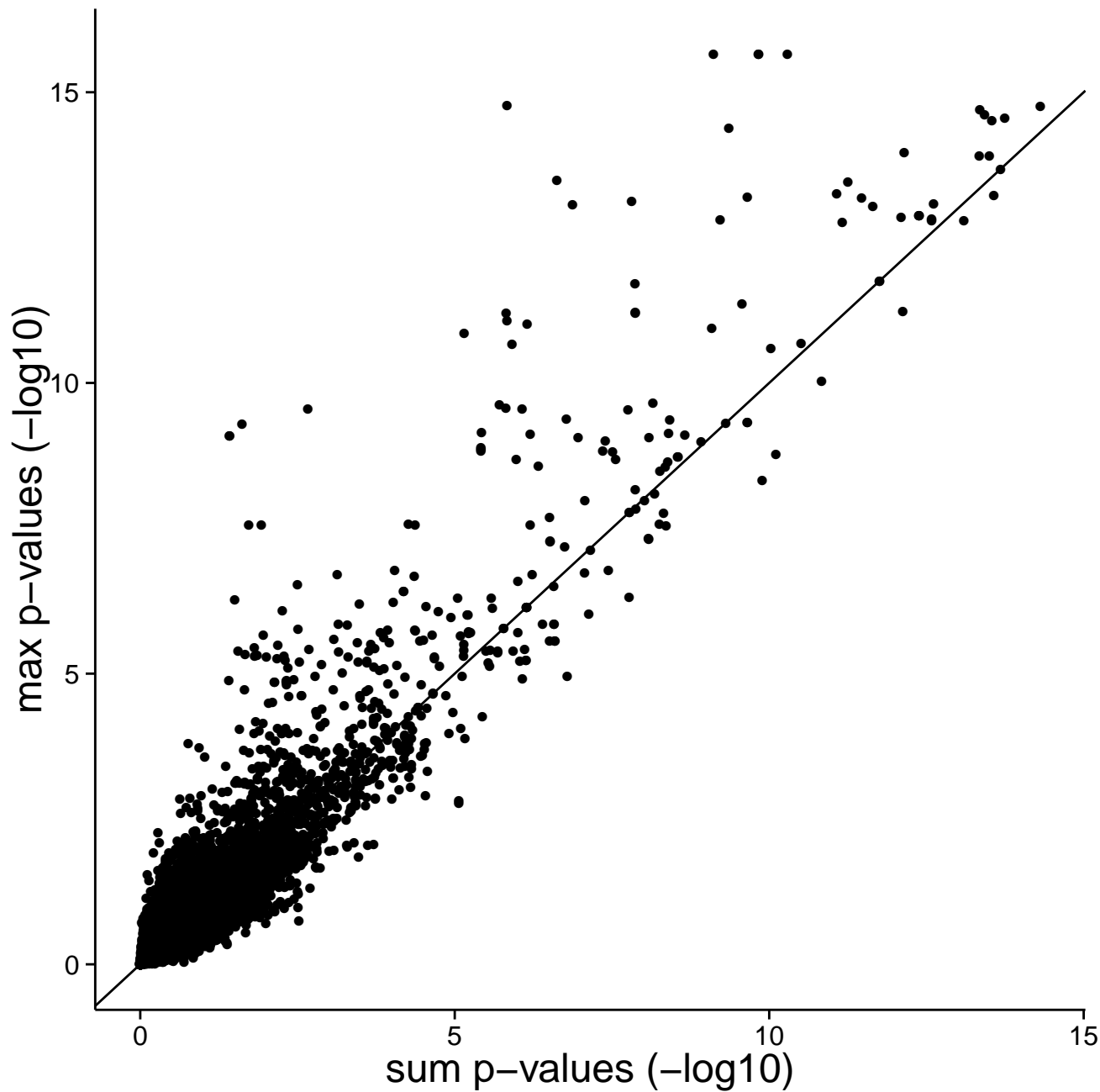

Supplement: S3 Fig — We compared max and sum gene scores directly for a large-scale blood HDL level meta-analysis. Only gene scores up to 10−15 are displayed, which truncated 6 genes with very large max scores. R2 between the–log10-transformed variables is 90%. Max scores tend to be larger when the two methods do not agree. (PDF) [file pcbi.1004714.s003.pdf]

**a****No genes fused**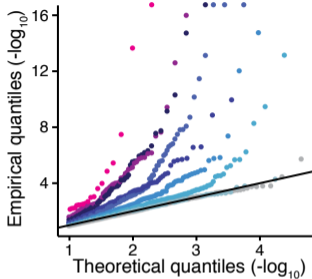**b****Proximal genes fused**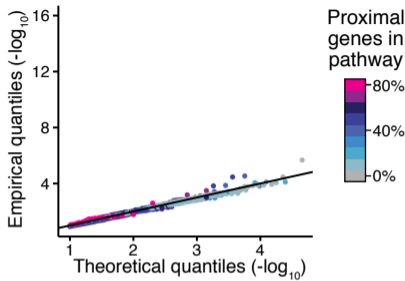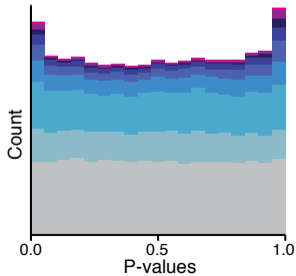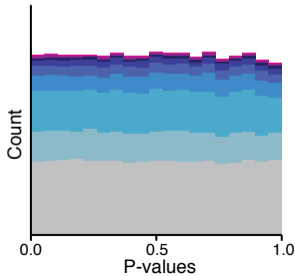

Supplement: S4 Fig — P-values for 1077 pathways from our pathway library were computed for 100 random phenotypes using the Pascal pipeline using max gene scores and chi-squared pathway integration strategy (a) without merging of neighbouring genes and (b) with merging of neighbouring genes (gene-fusion strategy). P-value distributions are represented by QQ-plots (upper panels) and histograms (lower panels). Results are colour-coded according to the fraction of genes in a given pathway that have a neighbouring gene in the same pathway, i.e. that are located nearby on the genome (distance <300kb). (a) P-values of pathways that contain genes in LD are strongly inflated without correction. (b) The gene fusion approach provides well-calibrated p-values independently of the number of pathway genes in LD. (PDF) [file pcbi.1004714.s004.pdf]

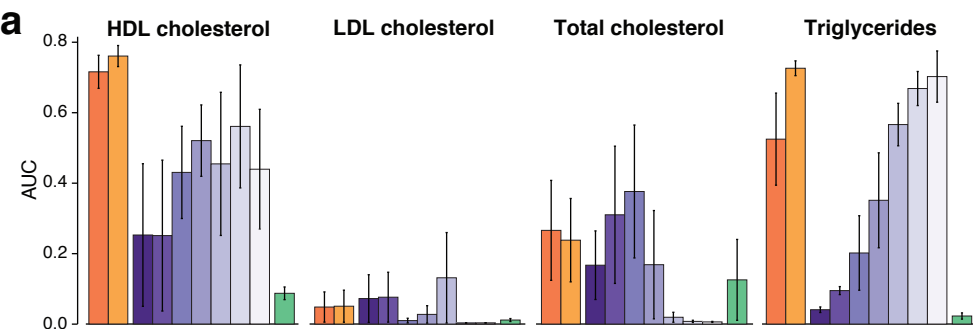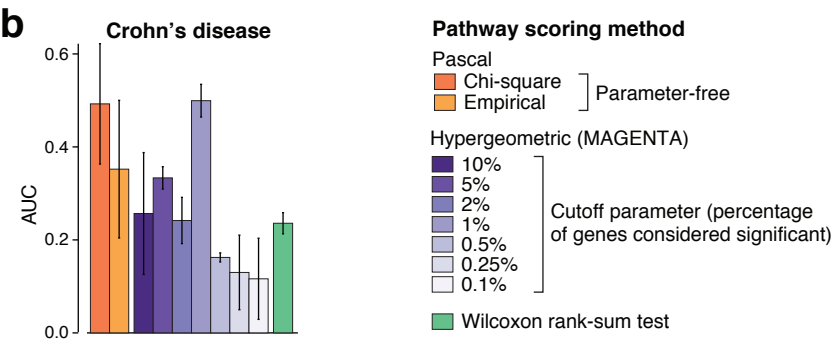

Supplement: S5 Fig — Displayed is the mean area under the precision-recall curve (AUC) for pathways identified using Pascal, a standard hypergeometric test at various gene score thresholds, and a rank-sum test (vertical bars show the standard error). We show results for the SOCS gene scores (MOCS gene score results are similar, see Fig 4 in the main text). a) Results for four blood lipid traits. A reference standard pathway list was defined as all pathways that show a significance level below 5×10^−6^, for any of the tested threshold parameters for hypergeometric tests in the largest study of lipid traits to date. The significance level of 5×10^−6^ corresponds to the Bonferroni corrected, genome-wide significance threshold at the 0.5% level for a single method. For each phenotype, error bars denote the standard error computed from three independent subsamples of the CoLaus study (including 1500 individuals each). We see good overall performance of Pascal pathway scores, whereas results for discrete gene sets vary widely with the particular choice for the threshold parameter of hypergeometric test. b) Results for Crohn’s disease using the same approach as in (a). A reference standard pathway list was defined as all pathways that show a significance level below 5×10^−6^ for any of the tested threshold parameters for hypergeometric tests in the largest study of Crohn’s disease traits to date. We observe that the chi-squared strategy outperforms all other strategies in this setting, whereas performance of the hypergeometric testing strategy varies. (PDF) [file pcbi.1004714.s005.pdf]

**a**

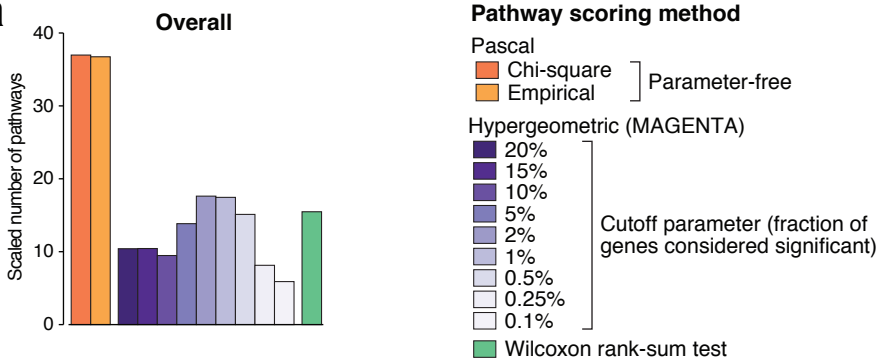**b**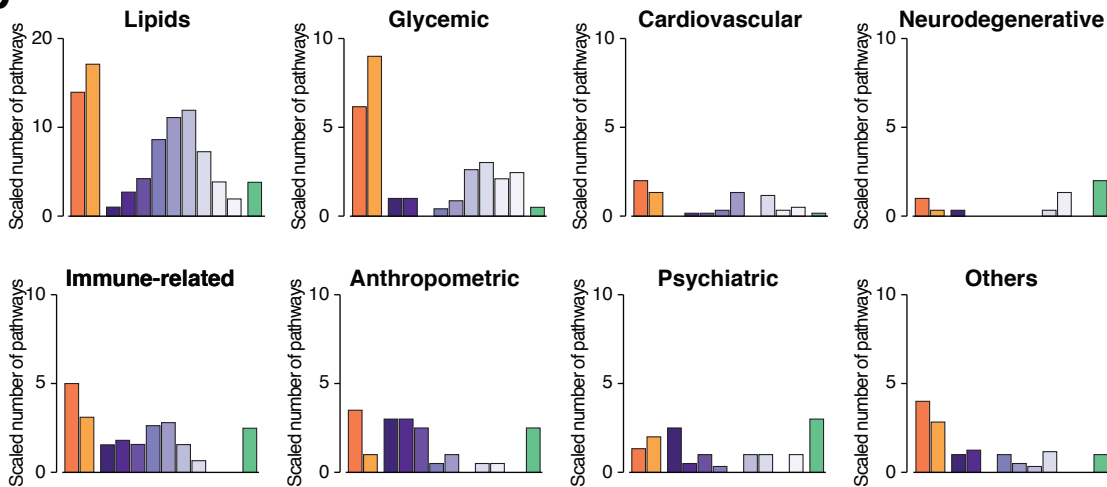

Supplement: S6 Fig — Bar heights represent the number of pathways found to be significant after Bonferroni correction. Within a given trait group, results are aggregated for all tested GWAS studies. 65 GWAS had at least one significant pathway in one of the tested method. For each GWAS, the raw number of significant pathways was divided by the number of pathways found by the best performing method. This was done to avoid that a few studies with many emerging pathways dominate. We show results for the SOCS gene scores (MOCS gene score results are similar, see Fig 5). (a) Results are aggregated over all trait groups. (b) Results for different trait groups. (PDF) [file pcbi.1004714.s006.pdf]

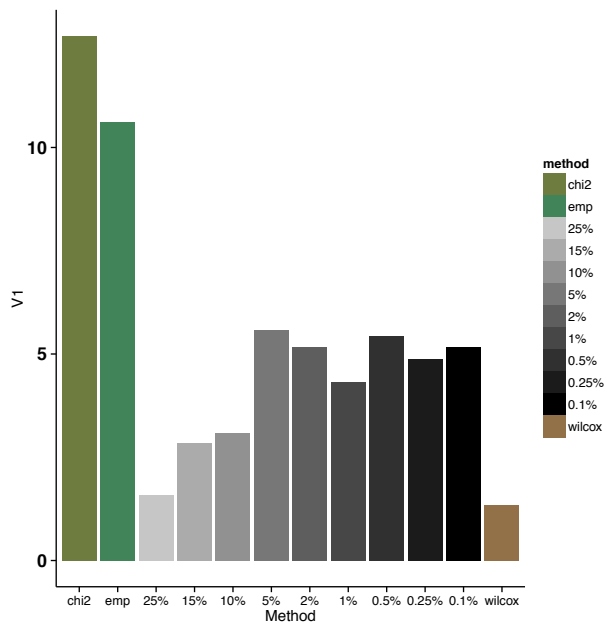

a)

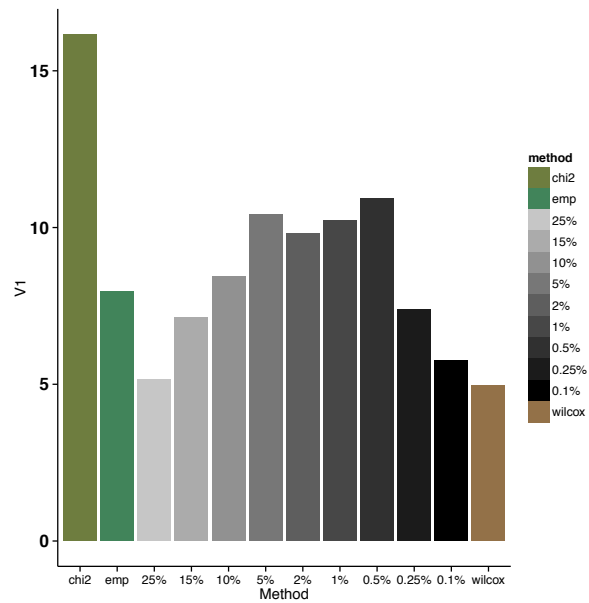

b)

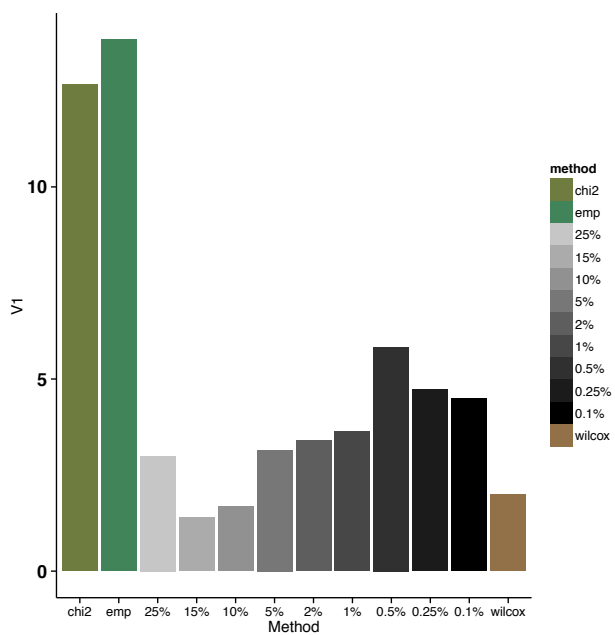

c)

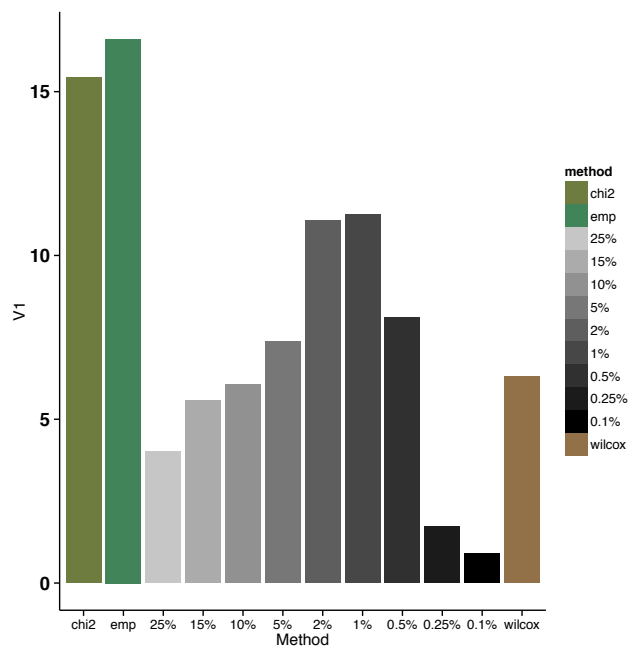

d)

Supplement: S7 Fig — Only GWAS studies for quantitative traits were used. Top panels (a,b) show results for max gene scores and bottom panels (c,d) show results for sum gene scores. (a,c) Results for all studies where the number of individuals was below 50,000. (b,d) Results for studies with sample sizes above 50,000. We see power gains in all cases. The improvements are particularly pronounced in lower powered GWAS. (PDF) [file pcbi.1004714.s007.pdf]

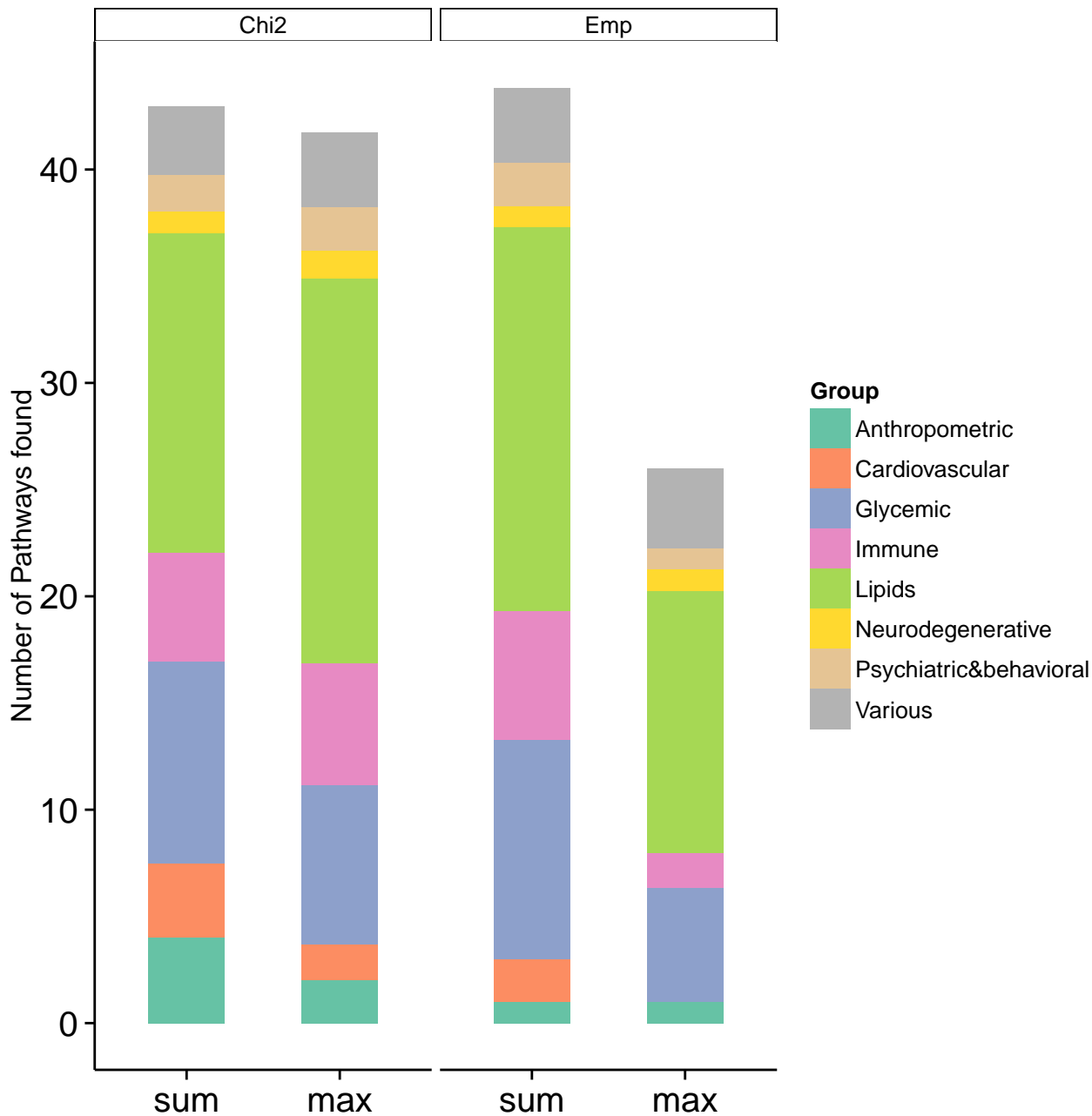

Supplement: S8 Fig — Bar heights represent the number of pathways found to be significant after Bonferroni correction. Within a given trait group, results are aggregated for all tested GWAS studies. For each GWAS, the raw number of significant pathways was divided by the number of pathways found by the best performing method. Results for SOCS and MOCS as well as the chi-square and empirical pathway scores are displayed. We observe a drop in performance for the combination of MOCS gene scores with empirical pathway scores. (PDF) [file pcbi.1004714.s008.pdf]

Scaled Number of Pathways

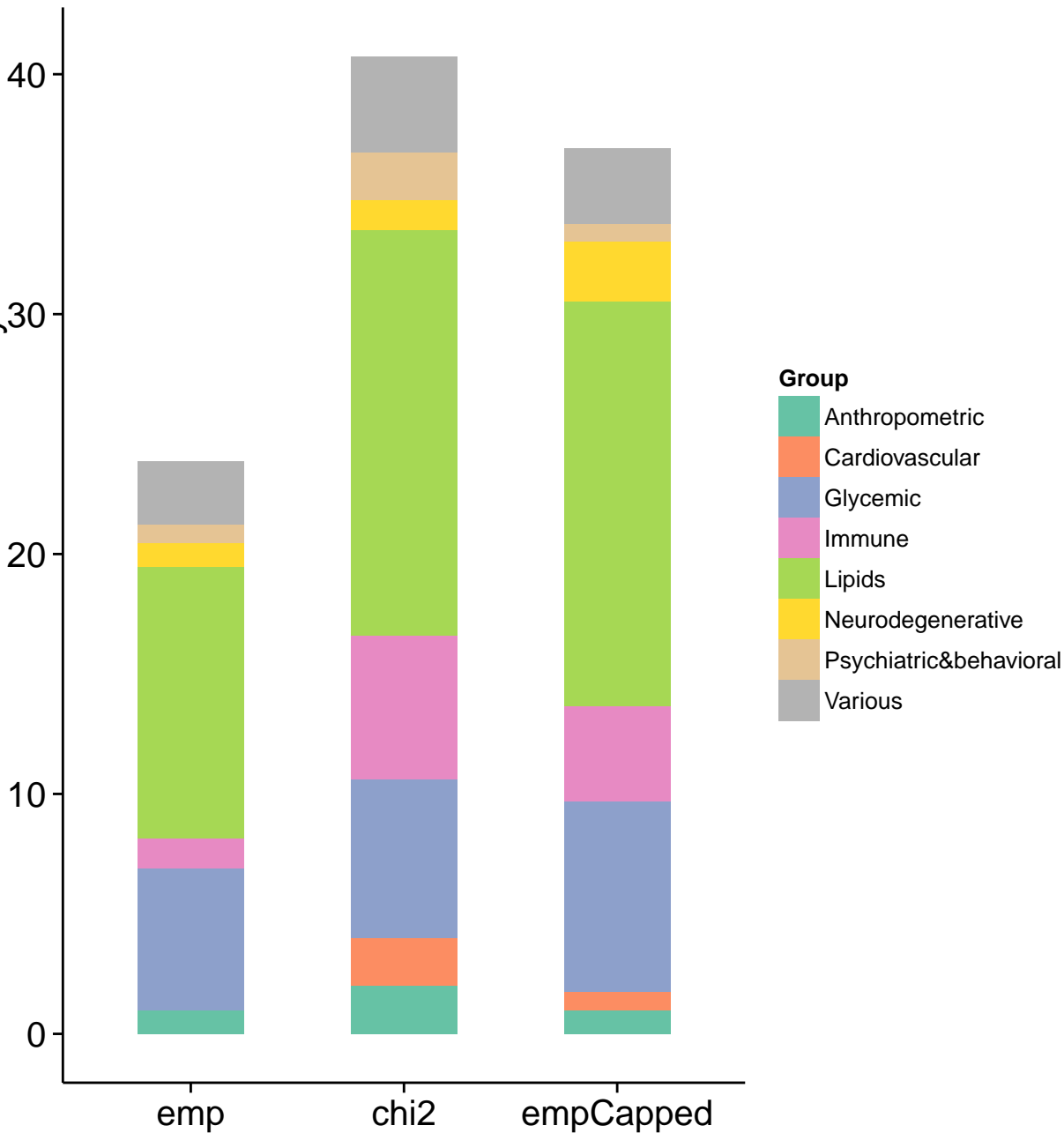

Supplement: S9 Fig — Bar heights represent the number of pathways found to be significant after Bonferroni correction. Within a given trait group, results are aggregated for all tested GWAS studies. For each GWAS, the raw number of significant pathways was divided by the number of pathways found by the best performing method. Max gene scores using empirical sampling pathway scores (emp) and chi-squared pathway scores (chi2) are compared to max gene scores combined with empirical sampling, where outlier gene scores (p-value <10−12) are set to 10−12 (empCapped). We chose the capping value such that the maximum–log10 p-value was roughly in the middle between genome wide significance threshold (8) and the maximum value that can be calculated for the sum statistic (15). (PDF) [file pcbi.1004714.s009.pdf]

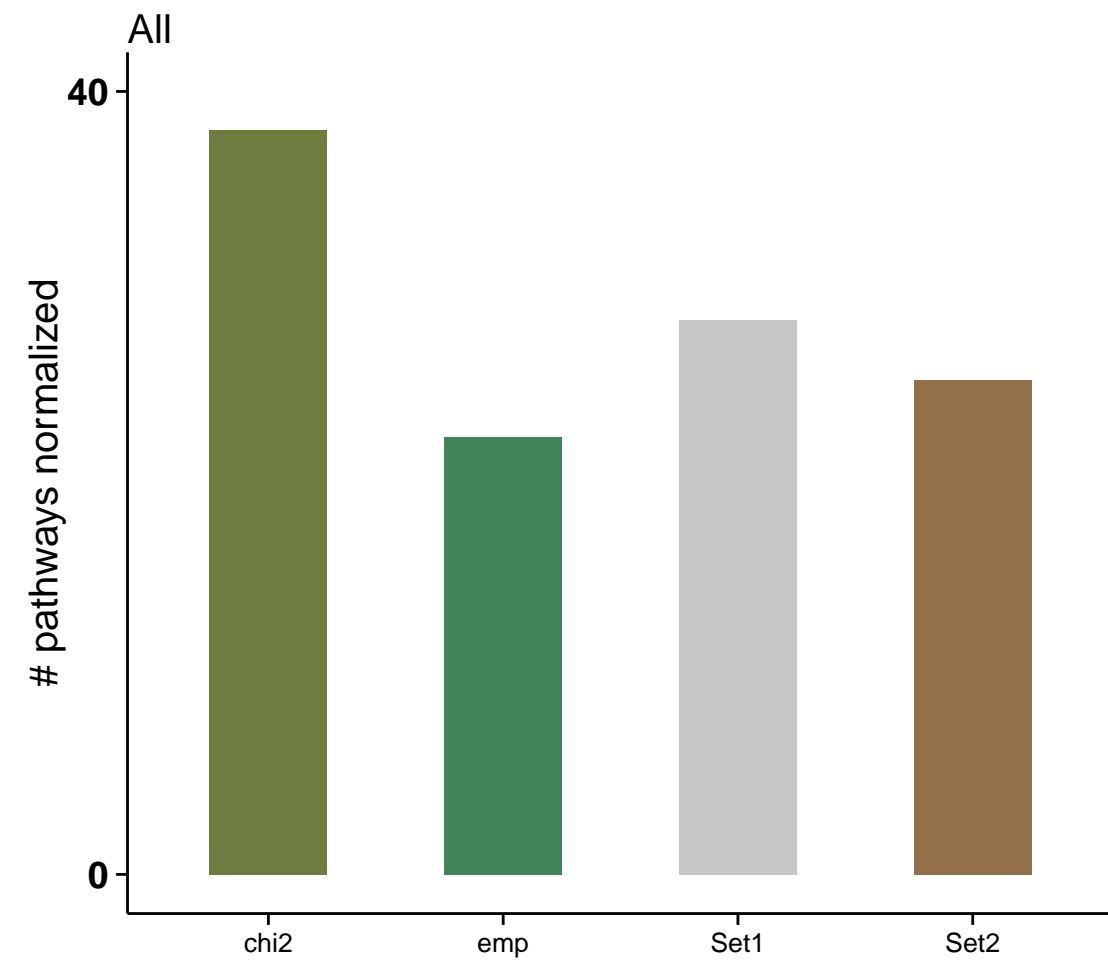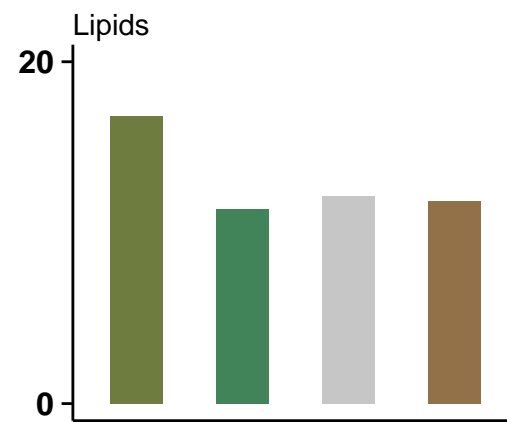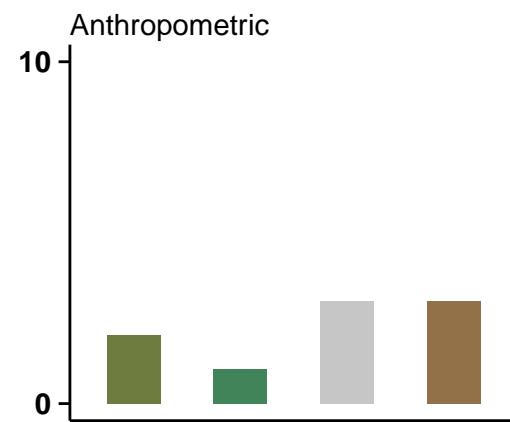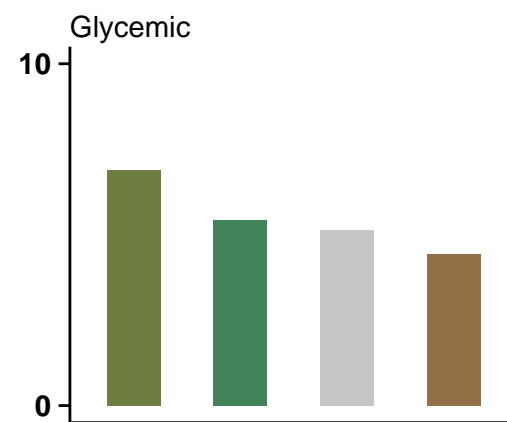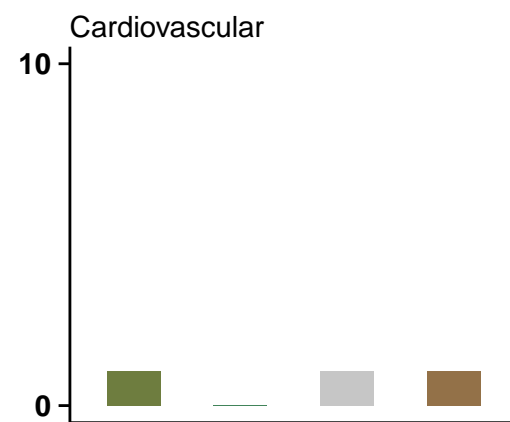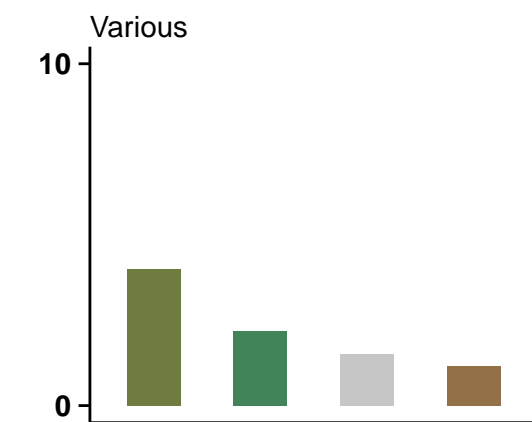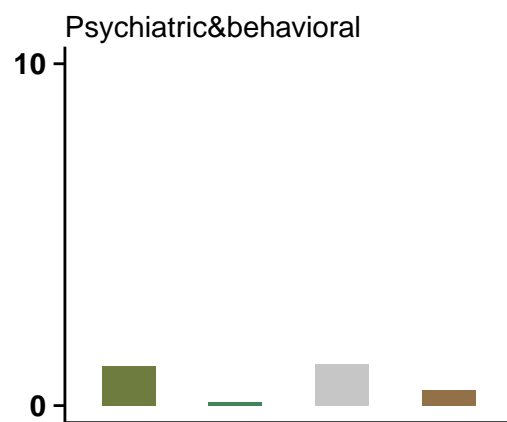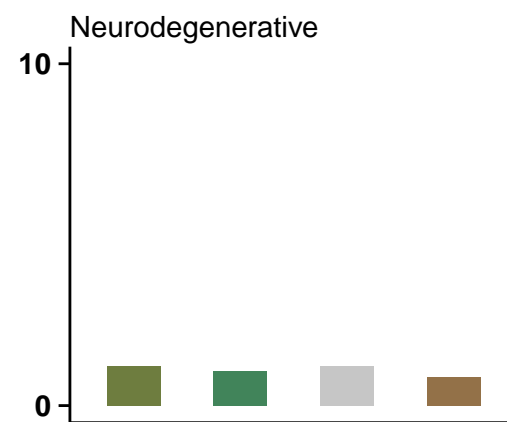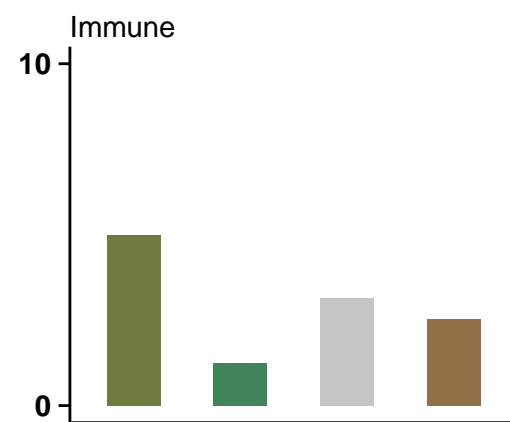

Supplement: S10 Fig — The same data as in Fig 5 is plotted here. However, instead of comparing Pascal pathway scoring methods with results for all hypergeometric threshold separately, we defined a new aggregated pathway score that picks the optimal threshold for each pathway over a range of hypergeometric threshold and correcting for the multiple number of tests by Bonferroni correction. Results for different sets of thresholds are displayed. Set1 refers to the complete set of thresholds (i.e.: 25%, 15%, 10%, 5%, 2%, 1%, 0.25%, 0.1%). Set2 refers to a set with thresholds more ‘spread out’ (i.e.: 25%, 5%, 1%, 0.25). We see that Pascal has better performance, except when combining the ‘empirical sampling’ pathway scoring method with max gene scores. (PDF) [file pcbi.1004714.s010.pdf]

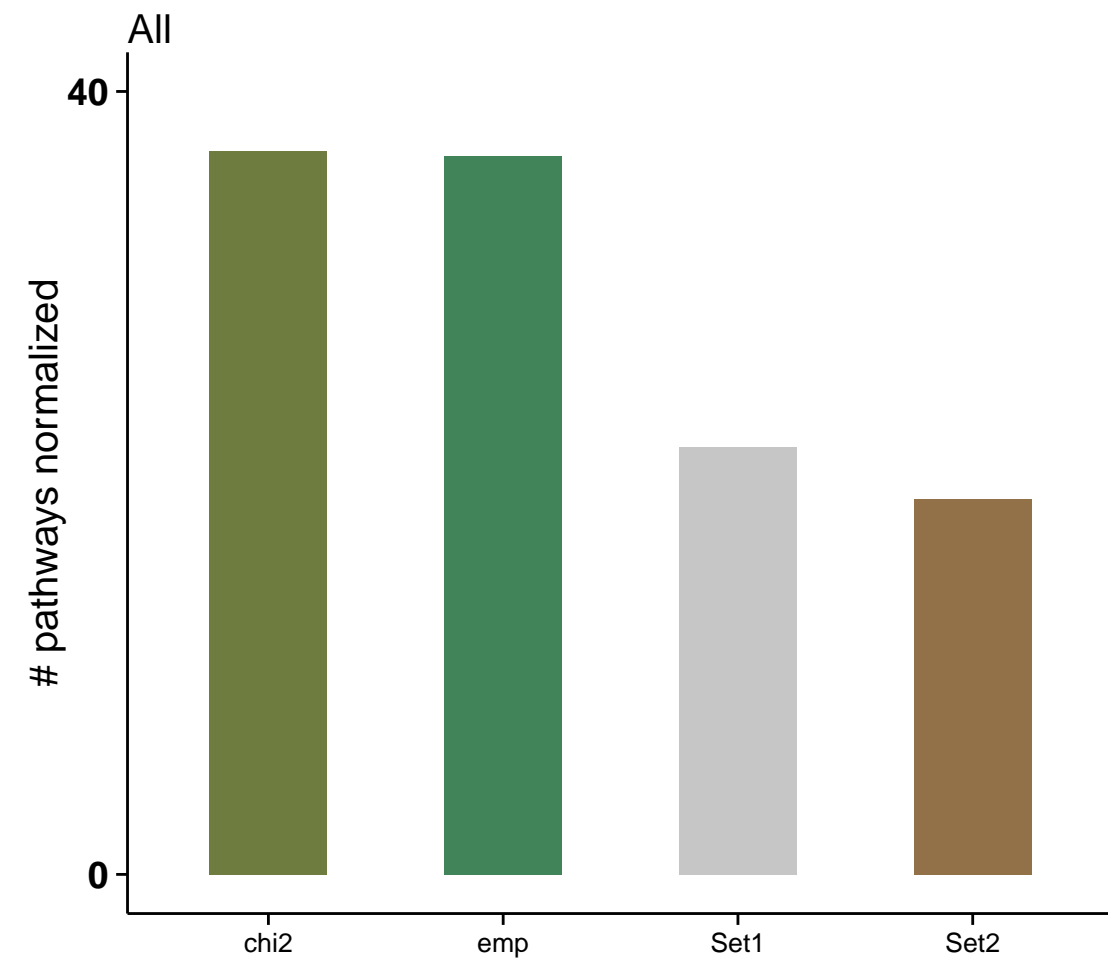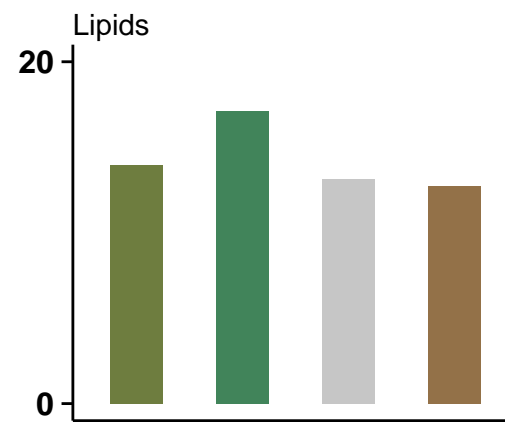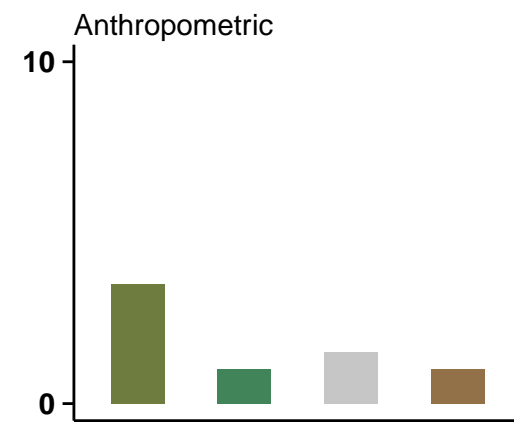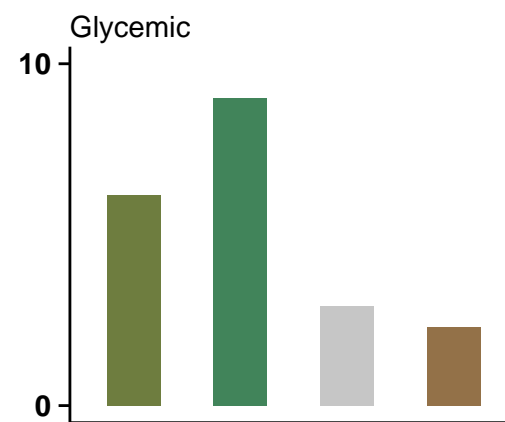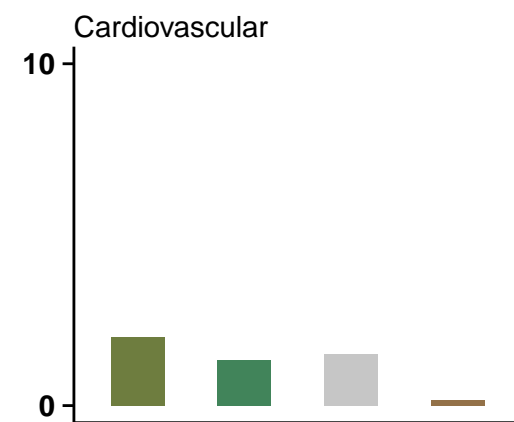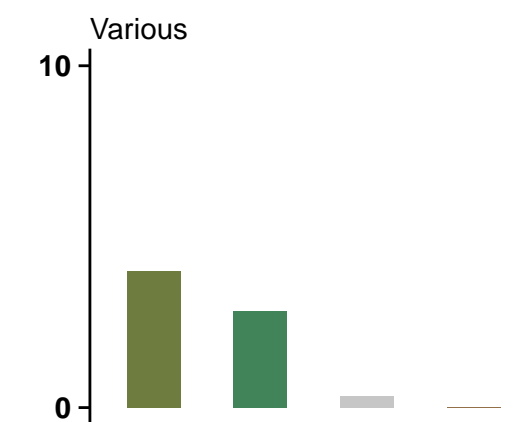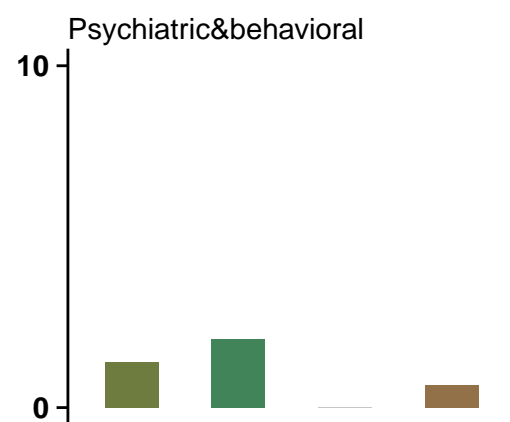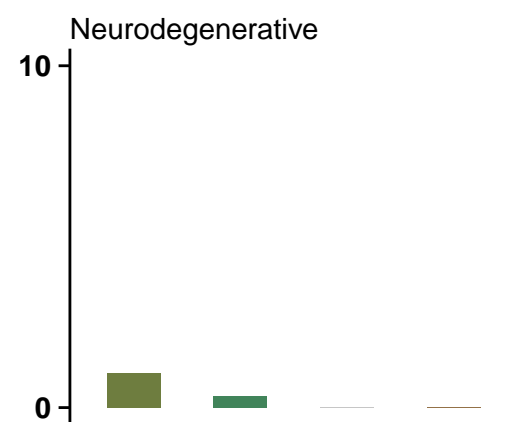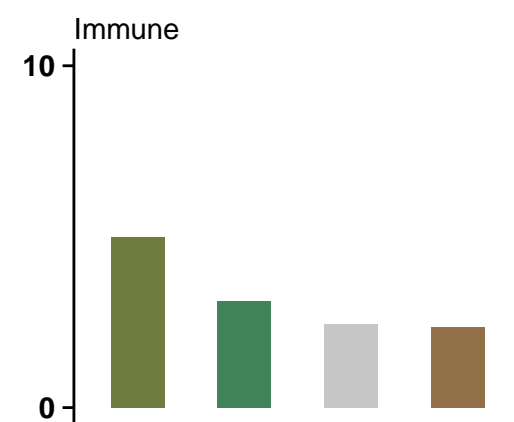

Supplement: S11 Fig — The same data as in Fig 5 is plotted here. However, instead of comparing Pascal pathway scoring methods with results for all hypergeometric threshold separately, we defined a new aggregated pathway score that picks the optimal threshold for each pathway over a range of hypergeometric threshold and correcting for the multiple number of tests by Bonferroni correction. Results for different sets of thresholds are displayed. Set1 refers to the complete set of thresholds (i.e.: 25%, 15%, 10%, 5%, 2%, 1%, 0.25%, 0.1%). Set2 refers to a set with thresholds more ‘spread out’ (i.e.: 25%, 5%, 1%, 0.25). We see that Pascal has better performance. (PDF) [file pcbi.1004714.s011.pdf]

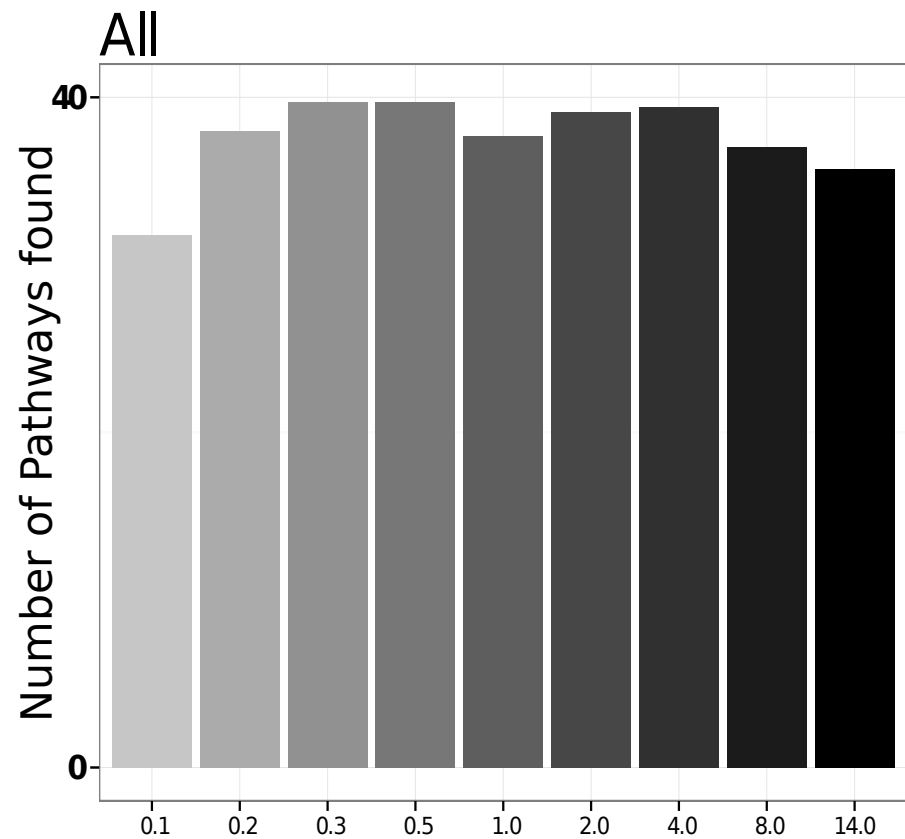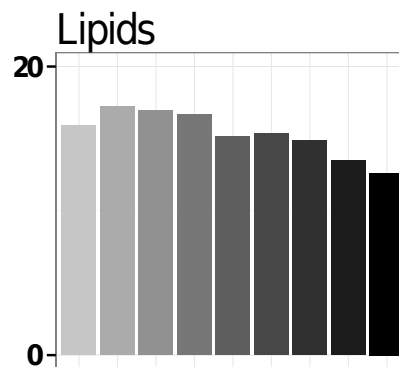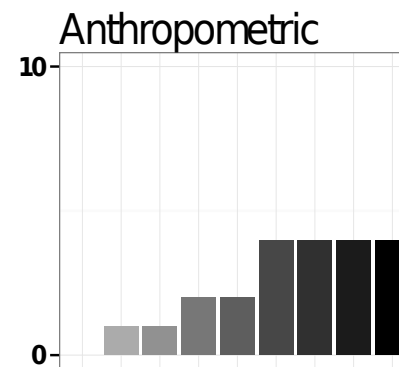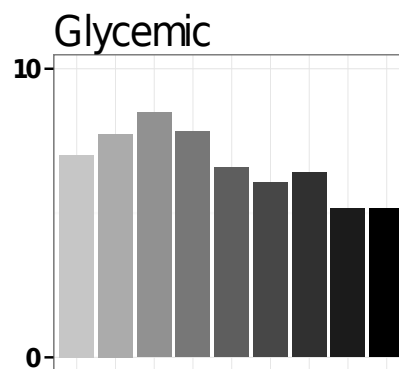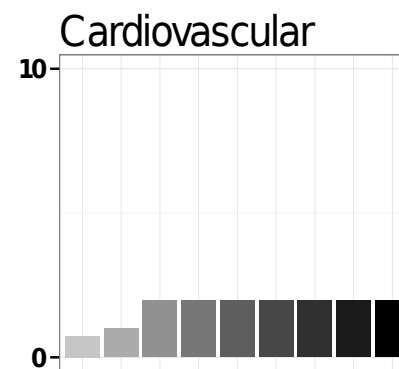

**Gamma shape parameter**

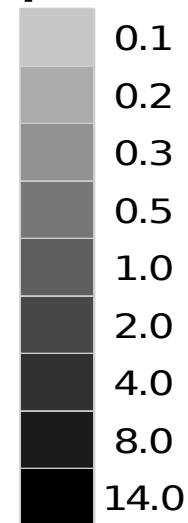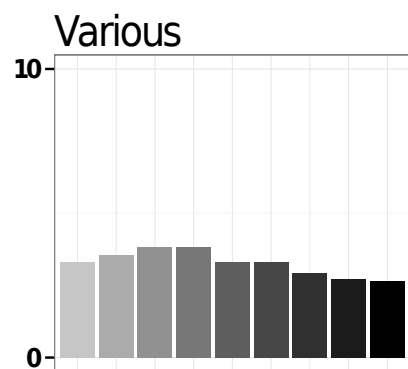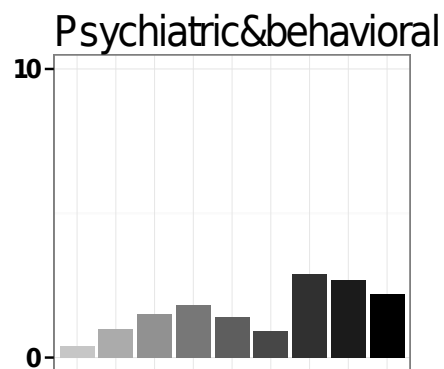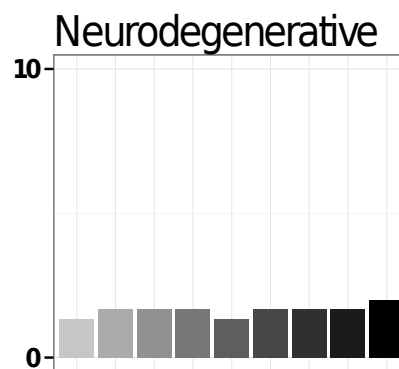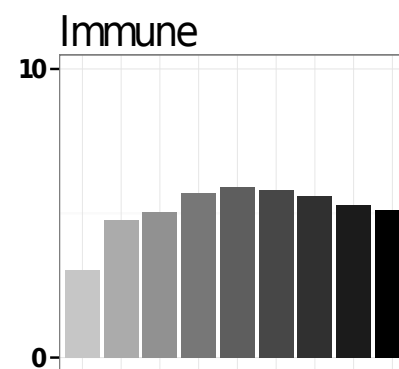

Supplement: S12 Fig — Bar heights represent the number of pathways found to be significant after Bonferroni correction. Different bars signify results for a different gamma shape parameter value. For each GWAS, the raw number of significant pathways was divided by the number of pathways found by the best performing method. Upper left panel ‘All’ refers to all traits stacked. We present here MOCS gene score based results. 52 GWAS showed at least one significant pathway in one of the evaluated scenarios. (PDF) [file pcbi.1004714.s012.pdf]

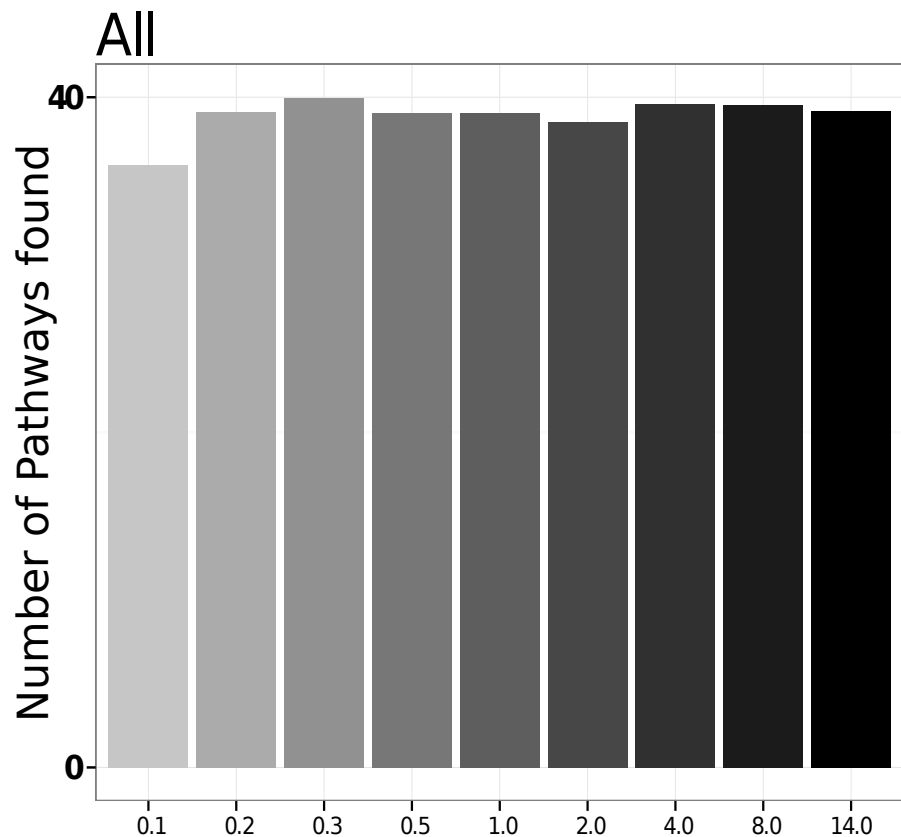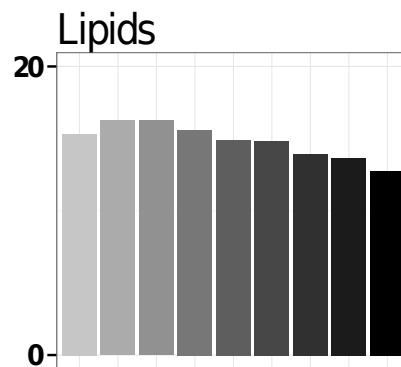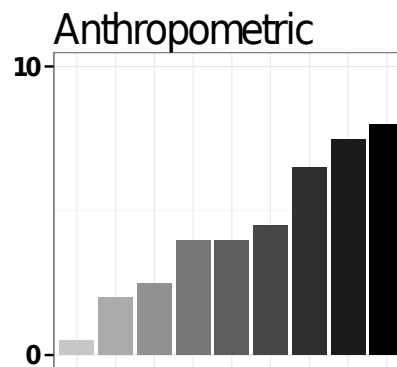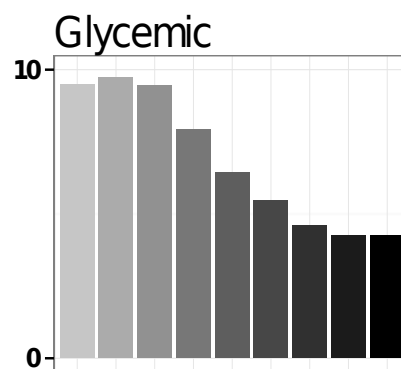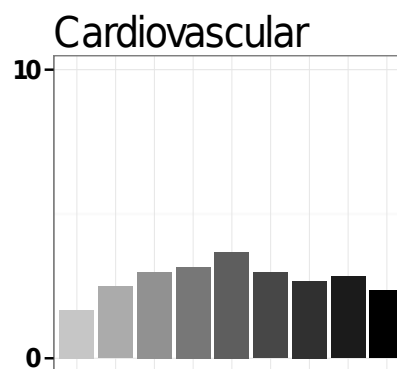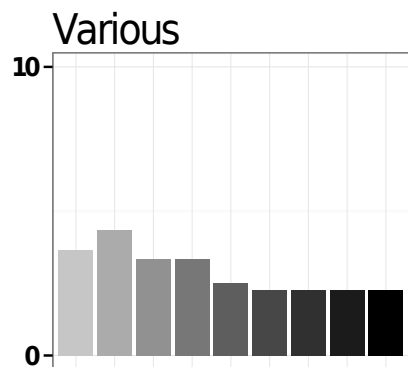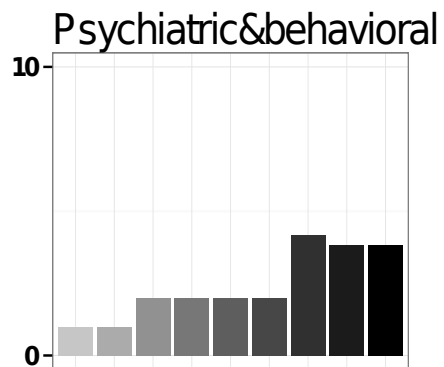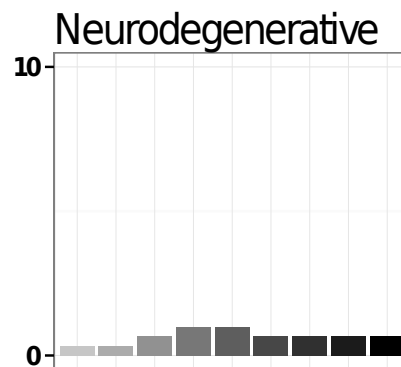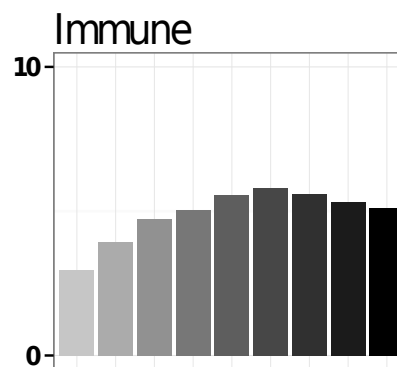

**Gamma shape parameter**

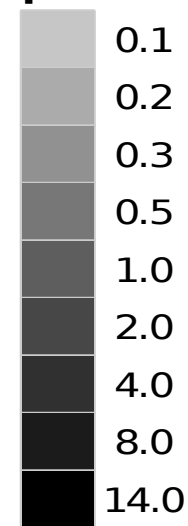

Supplement: S13 Fig — Bar heights represent the number of pathways found to be significant after Bonferroni correction. Different bars signify results for a different gamma shape parameter value. For each GWAS, the raw number of significant pathways was divided by the number of pathways found by the best performing method. Upper left panel ‘All’ refers to all traits stacked. We present here MOCS gene score based results. 60 GWAS showed at least one significant pathway in one of the evaluated scenarios. (PDF) [file pcbi.1004714.s013.pdf]

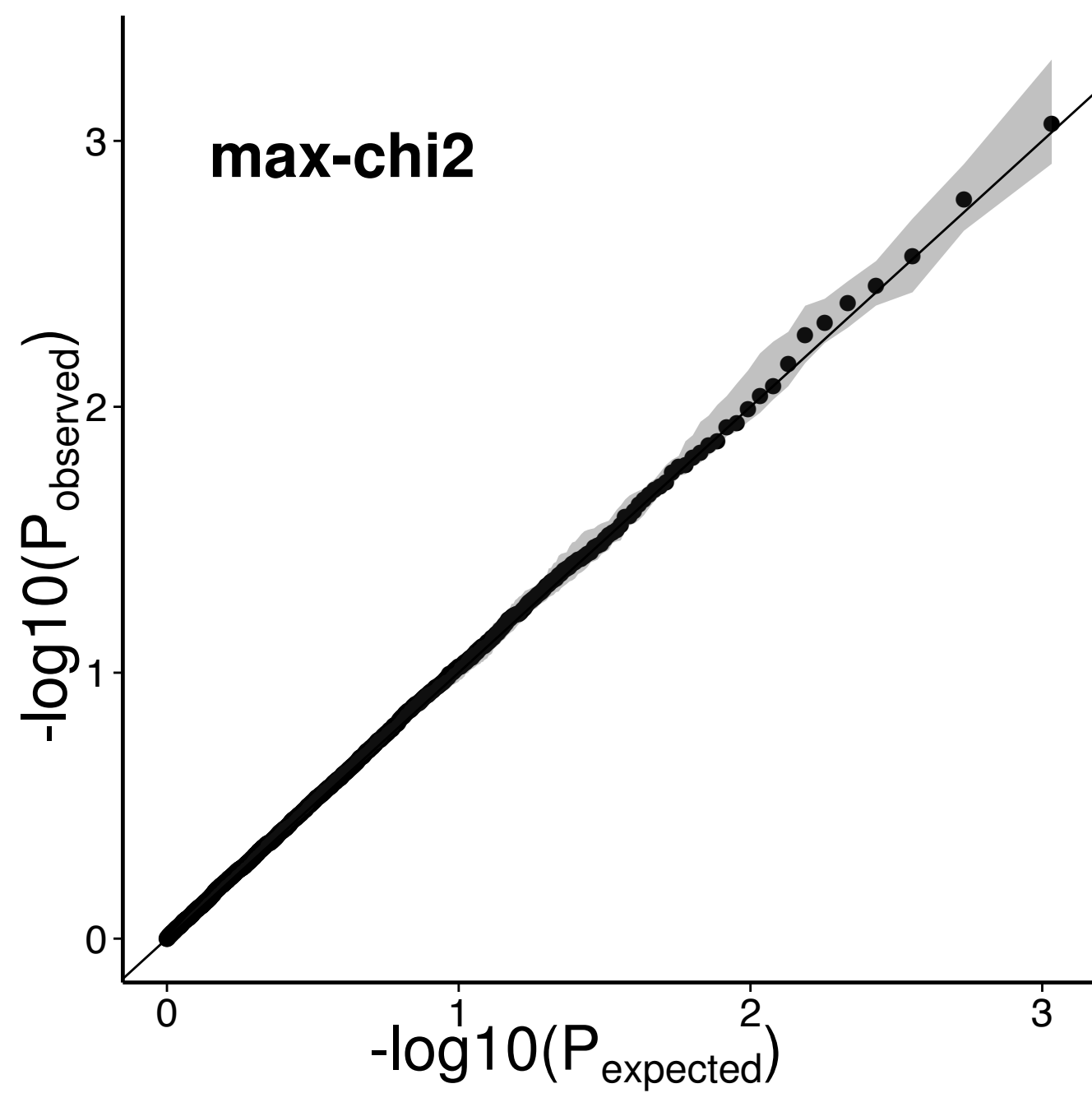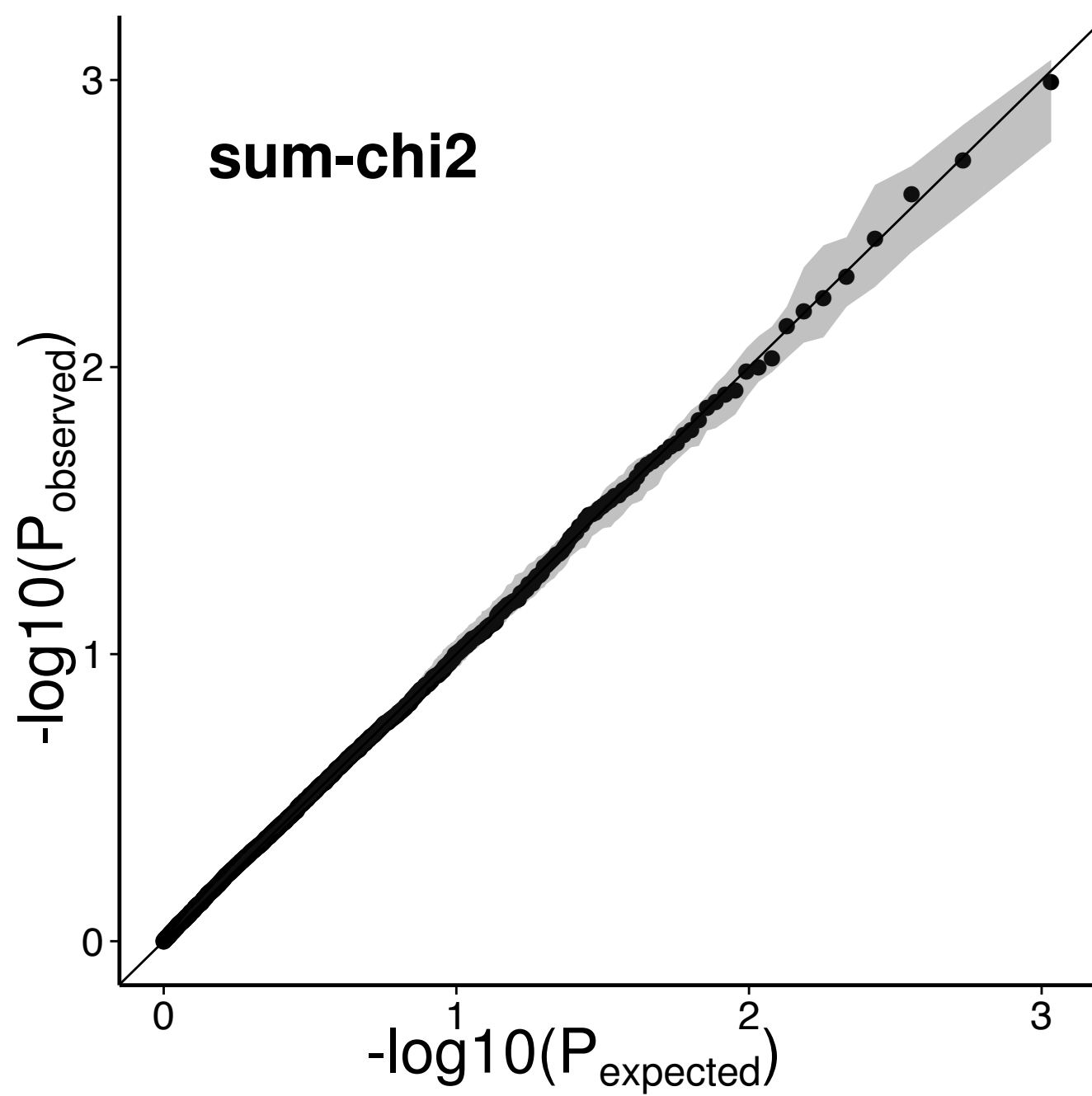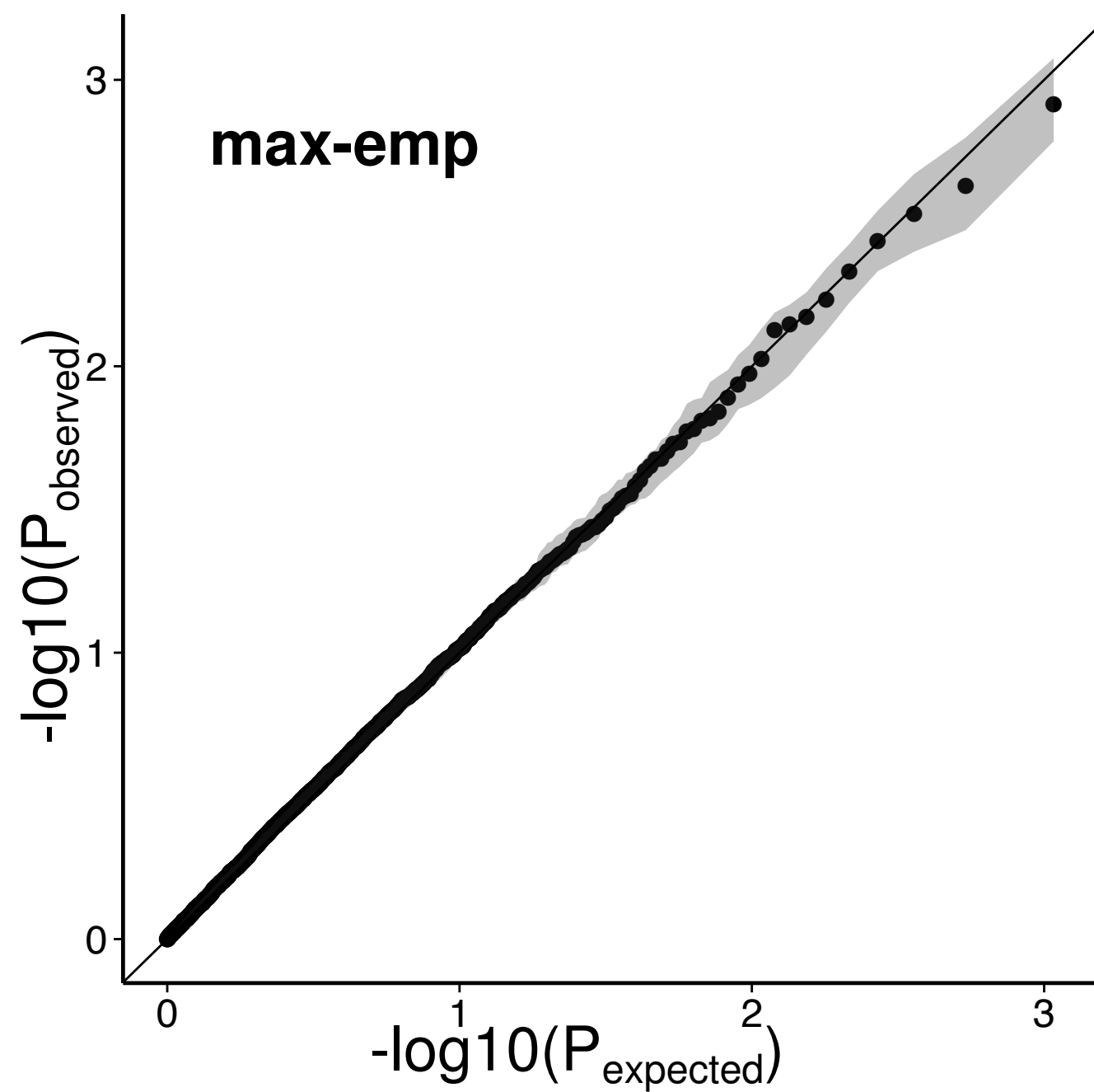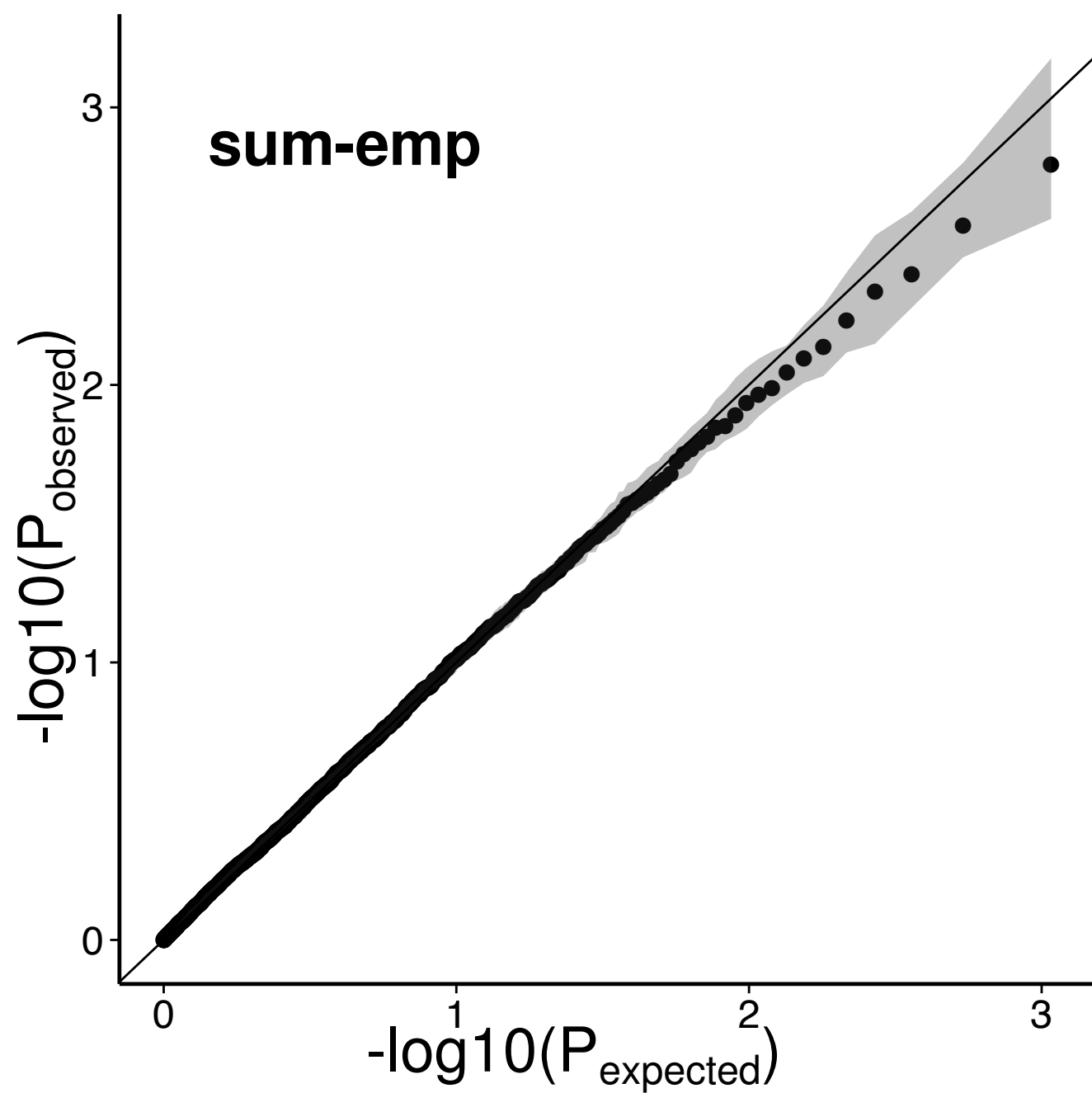

Supplement: S14 Fig — We first sampled 50 random SNPs assayed in CoLaus in or close to coding regions. Using the genotypes of the CoLaus study we then simulated phenotypes by adding up the sampled 50 SNPs with a normally distributed effect size with a variance of 0.04 plus Gaussian noise (with a variance of 1). We then ran GWAS for the simulated phenotype to obtain association summary statistics. The experiment was repeated 50 times. On average, this resulted in 18 independent, genome-wide significant gene score hits for each simulated GWAS (for the MOCS statistic). We applied Pascal to compute pathway scores for each of the 50 simulated GWAS. We found that the resulting pathway scores are well calibrated, i.e., they do not show inflation or deflation regardless of the setting used (max or sum gene score, chi2 or empirical enrichment test). The QQ-plots show the median value for each quantile across the 50 simulated GWAS. The shaded areas correspond to 95% confidence intervals for the median (estimated from 2000 bootstrap samples of size 50, with replacements). Similar results were obtained when varying the type and number of simulated causal SNPs and their effect size. (PDF) [file pcbi.1004714.s014.pdf]

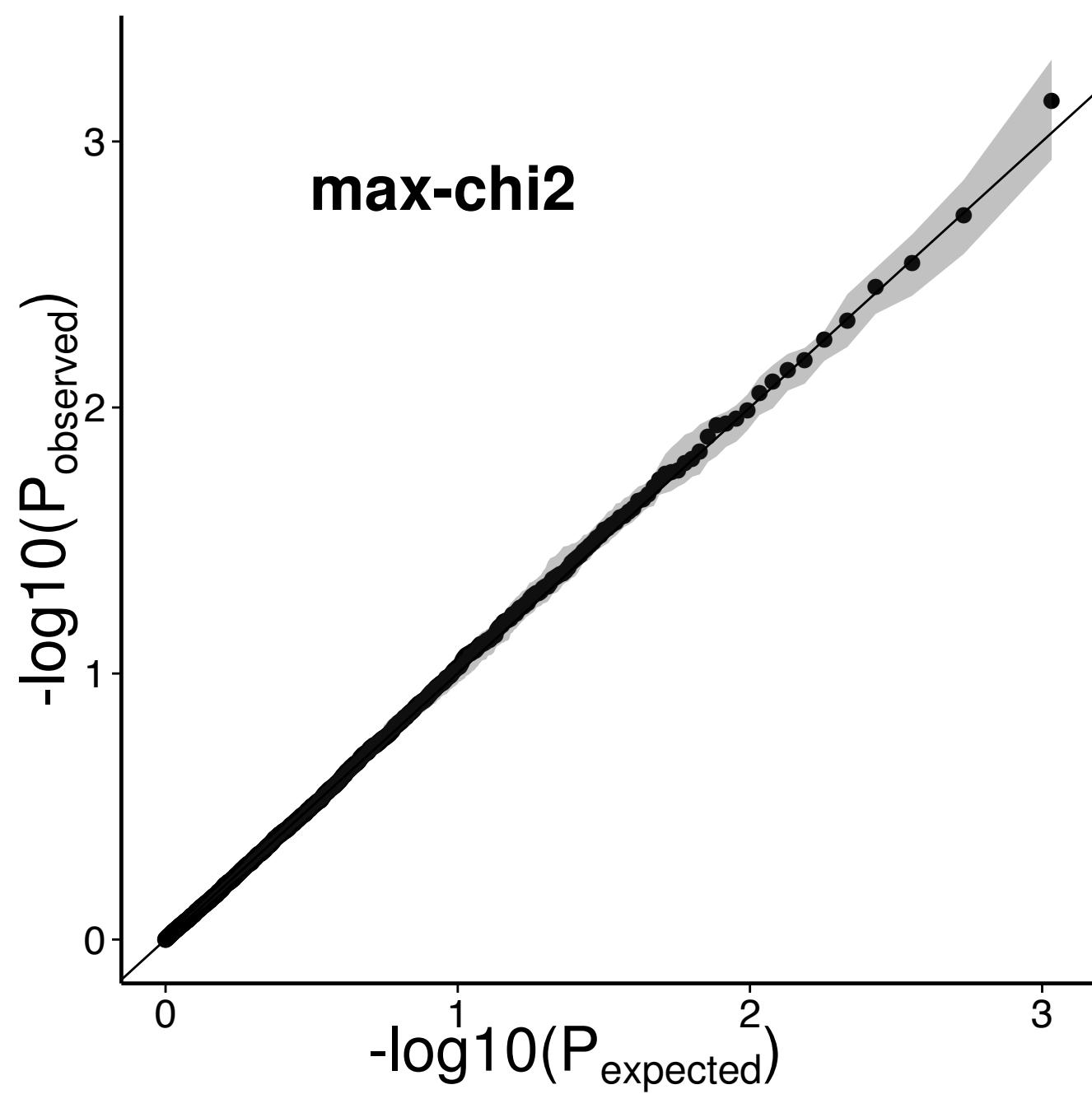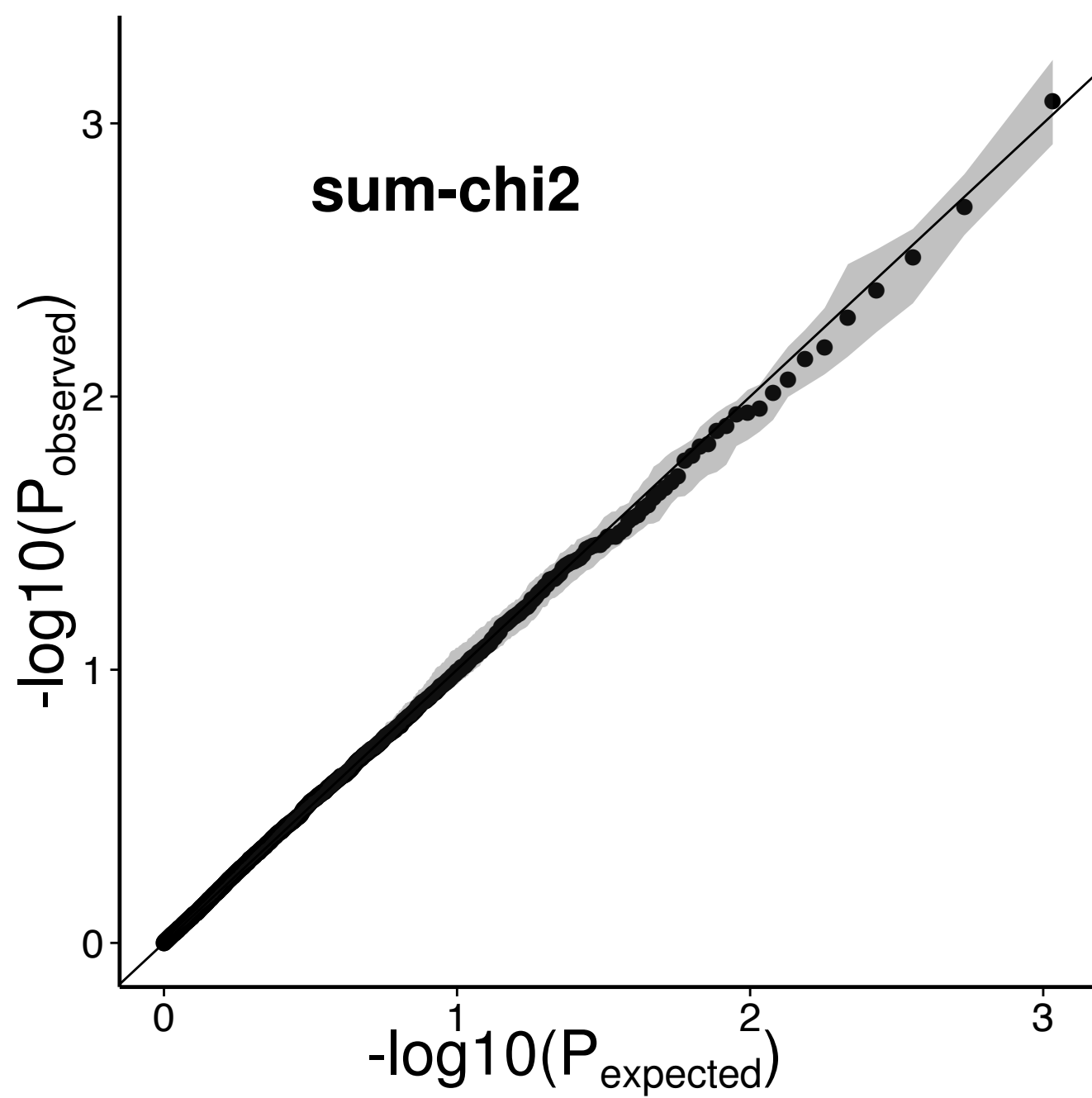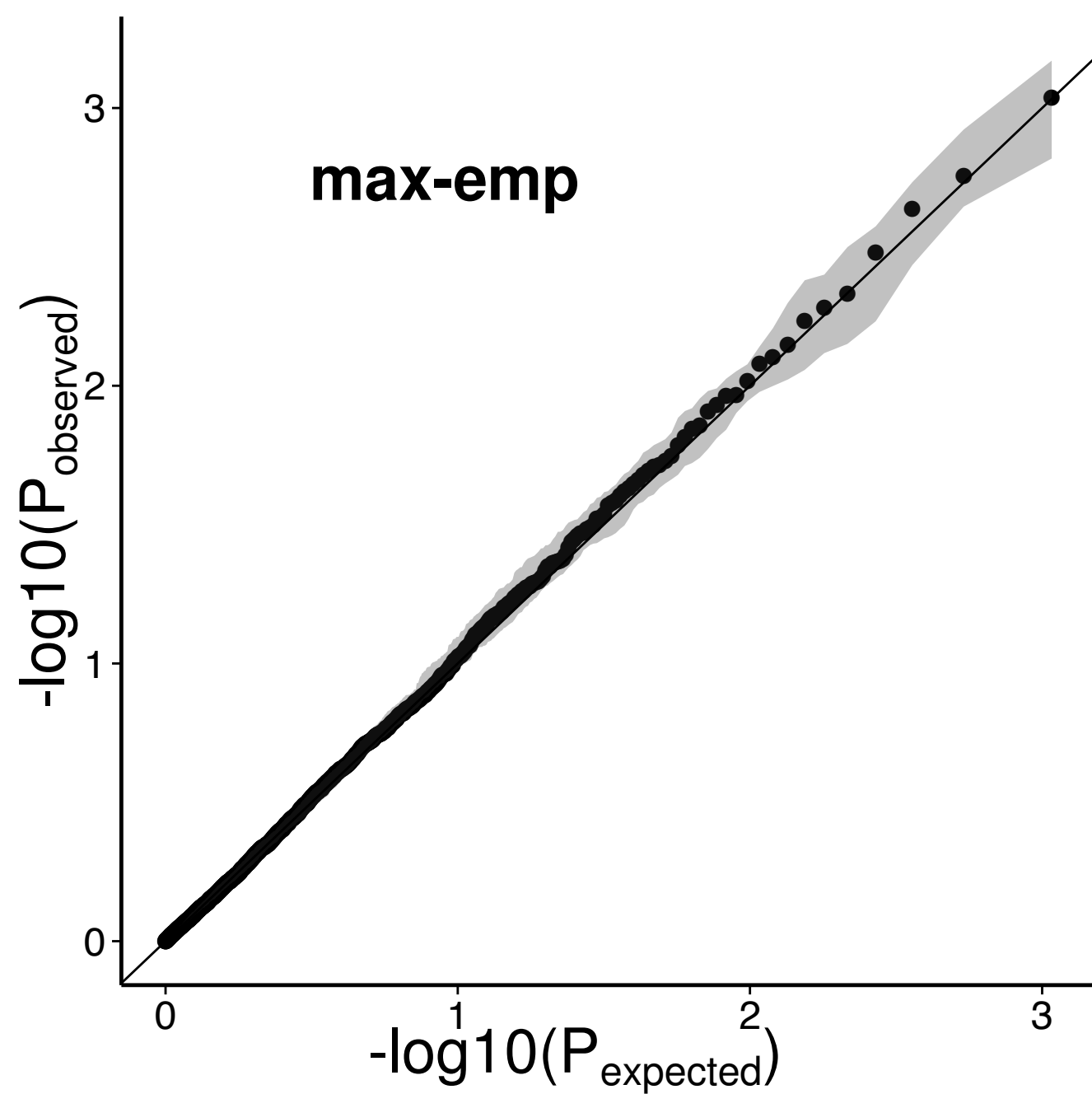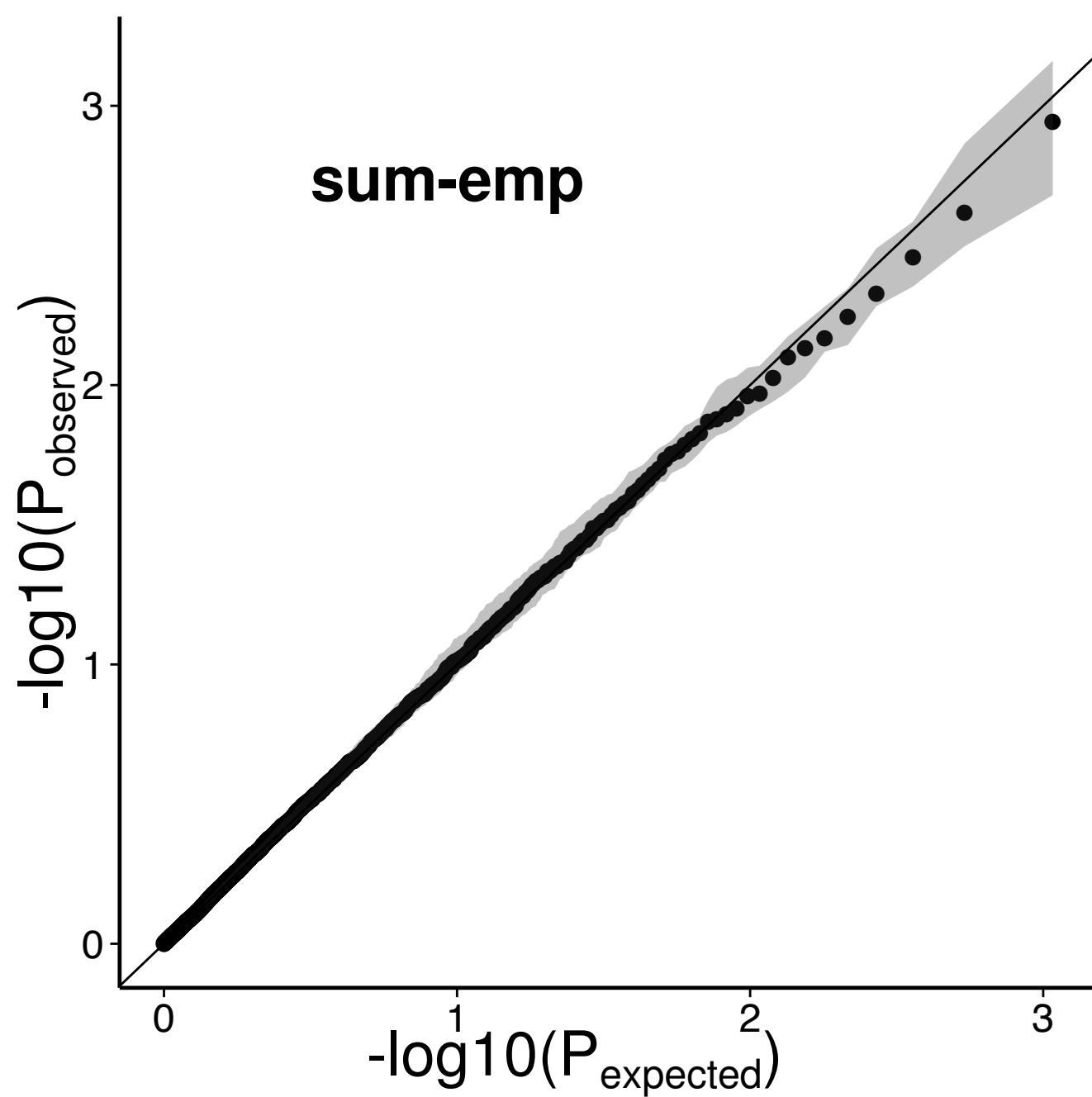

Supplement: S15 Fig — These QQ-plots correspond to an analysis equivalent to that of S14 Fig but with 50 SNPs chosen uniformly from all SNPs assayed in CoLaus, rather than from genic regions only. On average, this resulted in 12 independent, genome-wide significant gene score hits for each simulated GWAS (using the MOCS statistic). Note that this does not completely exclude the possibility of less well-calibrated scores in other settings. Deviations from perfectly calibrated scores may occur in the cases where true SNP associations are present, because the gene wise test statistic may have varying power for different genes depending on the genetic architecture of the associated phenotype and on certain gene properties (such as gene length, LD structure, SNP coverage, or SNP allele frequency). If a set of pathways contains many pathways enriched (or depleted) for genes with such confounding factors, inflation or deflation is possible. (PDF) [file pcbi.1004714.s015.pdf]
